# Association between the subcellular localization of host proteins and gut microbiome and metabolome in metabolic dysfunction-associated steatotic liver disease: a pilot study

**DOI:** 10.3389/fmolb.2026.1703547

**Published:** 2026-07-02

**Authors:** Shereen A. El Sobky, Nada El-Ekiaby, Injie O. Fawzy, Amira Khaled Abdelhamid, Heba Attia, Ibrahim H. Fayed, Yasser Badr, Mohammed Emadeldeen, Ahmed Nagy, Mohamed Negm, Mohamed Sherif Negm, Ahmed Moustafa, Mohamed El-Kassas, Mohamed A. Farag, Ramy K. Aziz, Ahmed I. Abdelaziz

**Affiliations:** 1 School of Medicine, Newgiza University (NGU), Giza, Egypt; 2 Department of Microbiology and Immunology, Faculty of Pharmacy, Cairo University, Cairo, Egypt; 3 Center for Genome and Microbiome Research, Faculty of Pharmacy, Cairo University, Cairo, Egypt; 4 Hepatology and Gastroenterology Department, National Hepatology and Tropical Medicine Research Institute, Cairo, Egypt; 5 Kasr Al-Ainy Viral Hepatitis Center (KAVHC), Kasr Al-Ainy University Hospital, Cairo University, Cairo, Egypt; 6 Clinical and Research Center for Intestinal Disorders, Cairo University, Cairo, Egypt; 7 Department of Pathology, Faculty of Medicine, Cairo University, Cairo, Egypt; 8 Biotechnology Graduate Program, American University in Cairo, New Cairo, Egypt; 9 Department of Biology, American University in Cairo, New Cairo, Egypt; 10 Endemic Medicine Department, Faculty of Medicine, Capital University (Formerly Helwan University), Cairo, Egypt; 11 Steatotic Liver Diseases Study Foundation in Middle East and North Africa (SLMENA), Cairo, Egypt; 12 Department of Pharmacognosy, Faculty of Pharmacy, Cairo University, Cairo, Egypt; 13 Healthcare Faculty, Saxony Egypt University (SEU), Badr City, Egypt

**Keywords:** MASLD, metabolome, microbiome, multi-omics, transcription factors

## Abstract

**Background:**

Metabolic dysfunction-associated steatotic liver disease (MASLD) is estimated to affect 38% of the global population, with limited options for treatment. It could progress to metabolic-associated steatohepatitis (MASH), fibrosis, and hepatocellular carcinoma. Agonists for farnesoid X receptor (FXR), peroxisome proliferation-associated receptors (PPARs), and sirtuin1 (SIRT1) are currently investigated for MASLD treatment. The subcellular localization of those proteins was shown to affect their function and could possibly be affected by different metabolites. Moreover, while those protein targets were found to be affected by the gut microbiome in mice, they have not yet been investigated in humans. Existing evidence independently links the gut microbiome to MASLD onset and demonstrates that host proteins are impacted by the microbiome. Therefore, we aimed at using integrative multi-omics analysis to investigate the interrelationship between the gut microbiome, fecal and serum metabolomes, and those selected protein targets in a cohort of patients with MASLD to identify potential markers differentiating MASLD and MASH.

**Methods:**

Serum and stool samples were collected from patients with MASLD and healthy controls, while formalin-fixed paraffin-embedded (FFPE) liver biopsies and clinical laboratory tests were obtained from patients only. Expression of the protein targets was analyzed by immunohistochemistry (IHC). Microbiome and metabolome analyses were performed, followed by bioinformatics, correlation, and multivariate and integrated multi-omics analyses.

**Results:**

SIRT1 and FXR subcellular localizations were correlated with multiple bacteria and metabolites, respectively. Three genera (*Rothia*, *Haemophilus*, and *Acetatifactor*) correlated with NAFLD activity score (NAS), and a signature of 20 bacterial genera, 10 fecal and 30 serum metabolites, and 3 host proteins differentiated between MASLD and MASH. Moreover, in silico analysis suggested myristic, lauric, octanoic, and nonanoic acids to putatively affect peroxisome proliferator-activated receptor alpha (PPARA) and FXR, and *Coprobacter* as an important contributor in our multi-omics model.

**Conclusion:**

Our data suggest bacteria and metabolites which potentially affect the subcellular localization, and hence activity, of anti-lipogenic proteins in MASLD patients. We also propose novel discriminatory markers between MASLD and MASH. Our findings form the groundwork for future mechanistic studies of both host and microbial factors possibly contributing to the multifaceted disease outcome and offer potential diagnostic markers.

## Graphical Abstract

**Figure:**
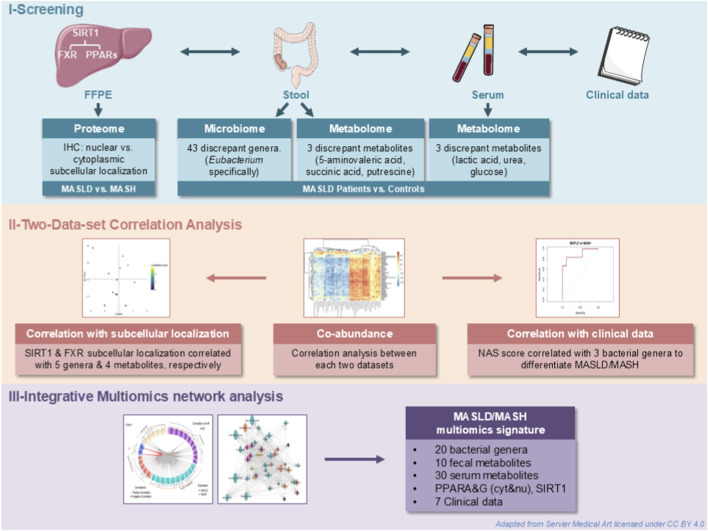
Graphical abstract showing a summary of the workflow and findings. Serum and stool samples were collected from patients with MASLD and healthy controls, and formalin-fixed paraffin-embedded (FFPE) liver biopsies and clinical lab tests were collected from patients only. Expression analyses of the protein targets using immunohistochemistry (IHC) and microbiome and metabolome analyses were performed. Correlation analyses were performed between pairs of datasets. SIRT1 and FXR subcellular localizations were correlated with multiple bacteria and metabolites, respectively. Three genera (*Rothia*, *Haemophilus*, and *Acetatifactor*) correlated with the NAS. An integrative analysis was performed to investigate whether all players may correlate together and yielded a signature of 20 bacterial genera, 10 fecal and 30 serum metabolites, and three host proteins as potential markers to differentiate between MASLD and MASH. Moreover, in silico analysis suggested myristic, lauric, octanoic, and nonanoic acids to putatively affect PPARA and FXR, and *Coprobacter* as an important contributor in our multi-omics model.

## 1 Background

Metabolic dysfunction-associated steatotic liver disease (MASLD) is a new term for non-alcoholic fatty liver disease (NAFLD) (Rinella et al., 2024; Tacke et al., 2024), a disease estimated to affect 38% of the adult population globally (Lin et al., 2025). It is defined as excessive hepatic triglyceride accumulation in the presence of at least one cardiometabolic risk factor (Tacke et al., 2024). Hence, MASLD encompasses the interplay between liver health and metabolic risk factors such as type 2 diabetes, obesity, dyslipidemia, and cardiometabolic risks (Yang and Song, 2023; Tacke et al., 2024). MASLD can progress to the more severe form, metabolic dysfunction-associated steatohepatitis (MASH), which is histologically characterized by hepatocellular ballooning and lobular inflammation (Tacke et al., 2024). It can further progress to fibrosis and cirrhosis (Mantovani et al., 2020) and is usually associated with cardiovascular and chronic kidney diseases and hepatic and extrahepatic malignancies (Tacke et al., 2024).

No pharmacological treatments for MASLD were available until resmetirom received FDA approval in 2024 (Keam, 2024). Several other candidates are being investigated in clinical trials (Yang Y. Y. et al., 2022), including agonists for sirtuin1 (SIRT1) and its downstream targets farnesoid X receptor (FXR) and peroxisome proliferation-associated receptor alpha and gamma (PPARA and PPARG) (Yang Y. Y. et al., 2022; Hu et al., 2024; Tian et al., 2024). Few studies have elucidated the relevance of the subcellular localization of these proteins to disease progression.

SIRT1 is a deacetylase and a regulatory protein with protective functions in the liver. Its deletion in mice models induces hepatic steatosis and inflammation (Purushotham et al., 2009), while its activation suppresses *de novo* lipogenesis (Hou et al., 2008; Ponugoti et al., 2010). SIRT1 subcellular localization affects its function and disease pathogenesis, especially oncogenesis (Byles et al., 2010; Song et al., 2014).

FXR, regulated by SIRT1, is a nuclear receptor that regulates bile acids (BAs) and lipid metabolism. In the liver, FXR reduces hepatic fatty acids (FAs) by regulating various lipogenesis-related transcription factors (TFs), including PPARA, thereby providing protection against MASLD (Pineda Torra et al., 2003; Clifford et al., 2021). FXR mainly functions in the nucleus, and its export to the cytoplasm leads to its acetylation and degradation. MASLD treatment with FXR agonists is hindered by FXR acetylation and cytosolic retention during inflammation, which can be overcome by combination treatment with a SIRT1 activator, which increases FXR import into the nucleus, increasing its hepatoprotective effects (Cui et al., 2023).

PPARs are ligand-activated TFs downstream of FXR and SIRT1 (Pineda Torra et al., 2003; Tian et al., 2024). Generally, PPARs predominantly localize to the nucleus and can shuttle to the cytoplasm, which affects their molecular functions as well as clinical disease outcome (Patel et al., 2005; Shao et al., 2020; Peng et al., 2021). PPARA is predominant in the liver, and ligand-binding localizes it to the nucleus, where it activates its target genes, reducing hepatic triglycerides and regulating inflammation (Wu et al., 2021). Hence, PPARA agonists improve outcomes related to steatosis, inflammation, and fibrosis in preclinical MASLD models (Pawlak et al., 2015). PPARG is also expressed in the liver and in adipose tissues; PPARG increases dietary FAs and glucose uptake and promotes lipogenesis and triglyceride synthesis and storage (Berthier et al., 2021). Therefore, evidence suggests that the subcellular localization of FXR, SIRT1, PPARA, and PPARG can greatly affect their function and hence MASLD pathogenesis.

On another note, several studies have implicated the dysregulation of the gut microbiome in MASLD (Yang and Song, 2023), suggesting its role in disease onset (Le Roy et al., 2013). Moreover, fecal microbiota transplantation (FMT) alleviated MASLD in high-fat diet (HFD)-fed mice (Zhou et al., 2017). An altered gut microbiome affects MASLD through altering the intestinal lumen metabolites (Vallianou et al., 2021) and increasing the intestinal barrier permeability (Rochon et al., 2024), with subsequent passage of metabolites into the portal vein, adding to MASLD progression (Liu et al., 2023). Interestingly, MASLD animal models showed an impact of the gut microbiome on host targets, such as SIRT1, FXR, PPARA, and PPARG (Chen et al., 2018; Wu et al., 2021; Nian et al., 2023). Gut bacteria and bioactive molecules were found to alter the subcellular localization of PPARG, leading to anti-inflammatory effects (Kelly et al., 2004; Avila-Roman et al., 2018). Additionally, the BA metabolite hyodeoxycholic acid attenuates MASLD by affecting PPARA nucleus-cytoplasm shuttling (Zhong et al., 2023). All this highlights the role of the gut microbiome and metabolome in regulating gene expression and subcellular localization of host proteins.

Previous studies have indicated that these proteins play a hepatoprotective role against MASLD and that their function is highly dependent on their subcellular localization. *In vivo* studies show that their nuclear-cytoplasmic shuttling can be affected by gut microbiota and metabolites, factors which have been independently linked to MASLD development. A potential link between the gut microbiome, its metabolites, and 38 host proteins was predicted bioinformatically in MASLD, suggesting PPARG and FXR to be among the affected host proteins (Oh et al., 2023). However, no study has concurrently correlated all factors in one human MASLD cohort. We hypothesize that the subcellular localization, and hence the function, of these hepatoprotective host proteins may be affected by the gut microbiome and the metabolome, thereby influencing MASLD disease outcome. Hence, we aimed to examine the gut microbiome and the metabolome, and their correlations with the subcellular localization of SIRT1, FXR, PPARA, and PPARG in MASLD patients. In addition, we aimed to identify a discriminatory multi-omics signature between MASLD and MASH. In this context, we identified multiple gut bacterial genera and metabolites correlated with the subcellular localization of SIRT1 and FXR, respectively. We also identified three bacterial genera correlating with the NAFLD activity score (NAS), and a signature of 20 bacterial genera, 10 fecal and 30 serum metabolites, and 3 host proteins differentiating between MASLD and MASH.

## 2 Methods

### 2.1 Patients and sample collection

Stool samples, FFPE biopsies, and 3 mL of serum in sterile vacutainers were collected from 21 patients with MASLD. The degree of hepatic fat, interface hepatitis, ballooning, and lobular necrosis was confirmed by histopathological analysis. NAS was determined according to Kleiner et al. (2005). Clinical data were also collected for each of the 21 patients (Supplementary Table S1). For control subjects, only serum and stool samples were collected from 14 healthy volunteers (Supplementary Table S2). A FibroScan analysis was performed to ensure that healthy volunteers did not have fatty liver. The age of the participants included in this study was from 18 to 65 years old. All MASLD stages were accepted except those with decompensated liver or at the advanced fibrotic stage F4. Patients with malignancies and viral infectious diseases were excluded, as well as pregnant women. None of the subjects used antibiotics prior to sample collection. All patients gave their written informed consent. All experiments were conducted under the ethical standards of the Declaration of Helsinki and were approved by the institutional review board of Capital University (formerly Helwan University) (10-2021).

### 2.2 Chemicals and reagents

Methoxyamine hydrochloride (cat. no. 89803), N-methyl-N-(trimethylsilyl)-trifluoroacetamide (MSTFA) with 1% trimethylsilyl chloride (TMSC) (cat. no. 69478), acetonitrile (99.8%), xylitol (an internal standard for relative quantification using GC/MS) (cat. no. X3375), pyridine (cat. no. 270407), and standard n-alkane mixture (C10–C40) (cat. no. 68281) were purchased from Sigma-Aldrich (St. Louis, Mo., United States).

### 2.3 Immunohistochemistry

Tissues from FFPE biopsies of patients with MASLD were sectioned onto TOMO slides (cat no. TOM-1190, Matsunami glass Ind., Japan) for IHC of the four protein targets. For deparaffinization and rehydration, slides were heated in the oven at 80 °C for 40 min. Slides were then passed three times in xylene (cat no. X1423-15, Greham Pharmaceuticals) for 5 min each, followed by passing twice in 100% ethanol (Chemajet, Egypt) for 5 min each, then twice in 96% ethanol for 5 min each, then twice in 70% ethanol for 5 min each, and then in distilled water briefly. For the heat-induced epitope retrieval and antigen detection, an EnVision FLEX High pH Kit (DAKO Agilent, cat no. K8000) was used, following the manufacturer’s instructions. Slides were first put in the working solution of the target retrieval solution (1:50) in a steam cooker for 30 min and then cooled at room temperature for 20 min. This was followed by immersing the slides in 1X wash buffer for 3 min and then blocking with 5% bovine serum albumin for 30 min in the case of PPARA and PPARG. Slides were then washed with 1X wash buffer and incubated with primary antibodies at 4 °C overnight (1:100 PPARA (Santa Cruz, cat no. sc-398394); 1:66 PPARG (Cell signaling, catalog no. 2435S); 1:50 FXR (Cell signaling, catalog no. 72105S); and 1:50 SIRT1 (Cell signaling, cat no. 8469S)). The slides were washed again with 1X wash buffer, followed by the addition of peroxidase for 3 min. The slides were washed again in wash buffer, and then horseradish peroxidase was added and incubated in the dark for 20 min. Samples were washed twice in 1X wash buffer, and then DAB-containing EnVision substrate working solution was added. Staining was stopped using tap water once the color was developed. The slides were washed again with wash buffer, immersed in hematoxylin for 3 min, and then washed with tap water for 5 min.

For the IHC scoring, four different independent observers judged the degree of nuclear or cytoplasmic expression of the four proteins, where the percentage of stained nuclei or cytoplasm and the intensity of staining were scored. Percentage of staining was scored as follows: 0 = negative, 1 = 1%, 2 = 2–10%, 3 = 10–50%, and 4 = >50%. Intensity was scored as follows: 0 = negative, 1 = weak, 2 = clear, and 3 = strong. The scores for the percentage and the intensity of stain were multiplied to give a score for the degree of nuclear or cytoplasmic expression for each protein for each patient. An average of the four obtained scores was then used for further analysis. To determine the staining intensity threshold and account for interobserver variability, each observer independently screened the stained FFPE tissues for each protein and assigned a score = 3 (strong) to the FFPE showing the highest intensity. All other staining scores for this protein were determined compared to that tissue. Interobserver variability (reliability analysis) was calculated for nuclear and cytoplasmic scores using a two-way mixed model in SPSS software. Interobserver reliability for IHC stains was high (nuclear PPARG = 0.954, nuclear PPAR = 0.954, nuclear FXR = 0.916, nuclear SIRT1 = 0.97, cytoplasmic PPARG = 0.981, cytoplasmic PPARA = 0.885, cytoplasmic FXR = 0.978, and cytoplasmic SIRT1 = 0.994), confirming the reproducibility of the IHC analysis. For the subcellular localization of each protein, its nuclear score was divided by its cytoplasmic score to calculate a localization score for each patient. This localization score was then used to build a partial least squares (PLS) model between localization scores of SIRT1, FXR, PPARA, and PPARG and the significantly correlated microbiome and metabolome.

### 2.4 Microbiome analysis

#### 2.4.1 Fecal DNA extraction and quantification

DNA from 35 patient and control stool samples was extracted using the QIAamp DNA Stool Mini Kit for microbial analysis (cat no. 56404, QIAGEN, Germany). The nucleic acid concentration and purity (260/280 and 260/230 ratios) of each sample were first determined using a NanoDrop spectrophotometer (Thermo Scientific, United States). Samples with adequate DNA concentrations and purity were further quantified using the Qubit dsDNA HS Assay Kit (Life Technologies, United States) and a Qubit® fluorometer (Life Technologies, United States) for subsequent DNA sequencing.

#### 2.4.2 16S rRNA amplicon sequencing

High-quality and concentrated DNA from each fecal sample was sequenced at The Egyptian Center for Genome and Microbiome Research, Cairo, Egypt, on an iSeq™100 platform (Illumina, United States). The 2 × 150 bp paired-end protocol was performed to generate an Illumina amplicon library, following the Illumina 16S rRNA library preparation protocol. Subsequently, the V3–V4 variable regions of the prokaryotic 16S rRNA gene were amplified under the following polymerase chain reaction (PCR) cycling conditions: 95 °C for 3 min, 25 cycles of 95 °C for 30 s, 52 °C for 30 s, 72 °C for 30 s, and a final extension at 72 °C for 10 min. The generated PCR products were subsequently purified with AmpureXP beads (Beckman Colter, United States) and eluted in 10 mM Tris elution buffer (pH 8.5). Afterward, dual indices and Nextera XT Index primers (cat.no. FC-131-1001, Illumina, United States) were attached under the following PCR conditions: 95 °C for 3 min, eight cycles of 95 °C for 30 s, 55 °C for 30 s, 72 °C for 30 s, and a final extension at 72 °C for 10 min. The concentration of each library was assessed in a Qubit® fluorometer (Life Technologies, United States), and DNA integrity was checked by agarose gel electrophoresis (in 1% agarose gels). Finally, equimolar amounts of the validated libraries were pooled and loaded in an Illumina iSeq™ 100 i1 Cartridge kit (cat. no. 20021533, Illumina, United States).

#### 2.4.3 Taxonomic assignment

Raw sequence reads obtained from the iSeq instrument had no overlap between amplicons generated by primer pairs. For this reason, Illumina’s BaseSpace software, unlike commonly used microbiome analysis tools, uses a proprietary method, which does not require sequence assembly, for taxonomic assignment. Illumina BaseSpace software (Version 7.1.0, Illumina, San Diego, CA, United States) was used against the available taxonomy databases (Greengenes_13 and DADA-RDP_18).

### 2.5 Metabolomics analysis

#### 2.5.1 Sample preparation

The stool sample preparation method for GC/MS was adapted from Erben et al. (2021), where 250 mg of stool was weighed on the same day of collection and 750 μL of cold ethanol/PBS (85:15) buffer was added. Stool samples were vortexed for 2 min and then homogenized for another 2 min. This was followed by centrifugation at 4 °C for 15 min at 15,000 × g. The supernatant was then collected and stored at −80 °C until GC/MS derivatization. Serum samples were centrifuged, collected, and stored at −80 °C until GC/MS derivatization.

For GC/MS derivatization, 100 μL of serum or fecal water was mixed with 5 μL xylitol (1 mg/mL in methanol, acting as an internal standard) and 200 μL of cold acetonitrile for protein precipitation. The mixture was then centrifuged at 13,000 rpm for 10 min. The supernatant was then dried/desiccated at 45 °C using a speed vacuum concentrator (Eppendorf, Germany). For metabolite derivatization, 50 μL of methoxyamine (20 mg/mL pyridine) was added to each sample residue, which was then dissolved and transferred into GC vials and incubated at 60 °C for 2 h. A second step of metabolite derivatization was performed by adding 70 μL of MSTFA with 1% trimethylchlorosilane (TMCS) and 70 μL pyridine to each sample, followed by incubation at 60 °C for 1 h. Samples were wrapped in parafilm and stored at 4 °C till GC/MS run the following day. Quality control (QC) samples were prepared for either serum or fecal water samples by pooling all samples together. A QC sample is made to ensure the validity of the sample preparation and detection method. An adequate QC sample was used in PCA plots. Its central positioning confirmed reproducibility.

#### 2.5.2 GC/MS analysis

GC/MS was performed in a Shimadzu GCMS-QP 2010 instrument (Shimadzu Corporation, Kyoto, Japan), where an Rtx-5MS capillary column (30 m × 0.25 mm i.d. × 0.25 µm film thickness, Restek, United States) and a split–splitless injector were used for chromatographic separation. Initial column temperature was set at 70 °C for 3 min, then programmed to 315 °C at a rate of 10 °C/min, and then finally kept constant at 315 °C for 6 min. The injector temperature was 280 °C, and helium was used as a carrier gas with a flow rate of 1.24 mL/min. Mass spectra were recorded under the following conditions: filament emission current = 60 mA; ionization voltage = 70 eV; ion source temperature = 180 °C; and interface temperature = 280 °C. Diluted samples (1% v/v) were injected in split mode (split ratio, 1:10), and the injection volume was 1 μL. A standard n-alkane mixture (C10–C40) was injected into the GC/MS during the analysis of each batch of samples for the purpose of detection and elimination of shifts in retention time.

#### 2.5.3 GC/MS metabolite identification

Raw data acquired from Shimadzu GCMS-QP 2010 were exported in *. NetCDF format. To deconvolute the measured mass spectra prior to the database search, an automated mass spectral deconvolution and identification system (AMDIS 2.64, NIST, Gaithersburg, Md., United States, www.amdis.net) was used. The retention index (RI) for each peak was calculated relative to the standard n-alkane mixture (C10–C40). Identification of metabolites was performed by mass spectra matching of each individual component against the reference spectra of the NIST Mass Spectral Library 2011 (National Institute of Standards and Technology, Gaithersburg, MD, United States) using the NIST Mass Search Program MS Search 2.0, with a matching score above 70%, as recommended by Frau et al. (2021).

#### 2.5.4 GC/MS data processing for multivariate data analysis

MS-DIAL data analysis software was used for metabolite profiling using peak alignment, matching, and identification following default parameters for GC/MS analysis. Metabolite MS signal abundance values were normalized to the amount of recovered internal standard (xylitol) in each sample prior to multivariate data analyses. Multivariate data analysis was performed using the program SIMCA-P Version 13.0 (Umetrics, Sweden). Principal component analysis (PCA) and orthogonal projection to latent structure–discriminant analysis (OPLS–DA) were performed. T2 and distance-to-model (DModX) tests were used to identify sample outliers and show whether a sample fell within a pre-defined range of variation. Outliers were removed, and *R* ^2^ and Q^2^ were calculated to assess the quality of the OPLS model. *R* ^2^ represents the goodness of the model’s fit, while Q^2^ represents the predictability of the model. The variables responsible for the segregation of samples on the score plot were identified from the S loading plot of the OPLS-DA model.

### 2.6 Statistical and bioinformatics analyses

Rstudio v2023.12.1.402 was used for statistical and bioinformatics analyses. The ropls R package v1.36.0 was used for PCA, OPLS, and PLS analyses, after outlier removal. For visualization of those plots, ggplot2 package v3.5.1 was used. For correlation analysis between datasets, the psych package v 2.4.6.26 was used to calculate correlation coefficients and their respective *p*-values, while the pheatmap package v1.0.12 was used for visualizing correlation matrices. The Caret package v6.0-94 was used for building logistic regression models. The pROC package v1.18.5 was used for ROC AUC curve generation, and the *p*-values for this analysis were computed by verification package v1.42. The R mixOmics package v6.28.0 was used for generating and visualizing sPLS and DIABLO analyses. Networks generated by DIABLO analysis were visualized by the Cytoscape software. Potential molecular targets of metabolites were predicted by the SwissTargetPrediction tool (http://swisstargetprediction.ch/).

### 2.7 Integrated multi-omics analysis

The DIABLO framework from the mixOmics R package was used for multi-omics and integrated analyses. The block link within the design matrix was balanced and set to 0.5, a value between classification and prediction task. In addition, a data driven weight was selected based on PLS analysis performed between each two data-sets, which showed correlations ranging from 0.56–0.87 (Supplementary Table S56), indicating that a design weight of 0.5 can be chosen. The global performance of the model was assessed by the “perf” function with the design matrix 0.5, validation = “Mfold,”, folds = 8, nrepeat = 50. The performance plot suggested one component for the final model and that Mahalanobis distance would be better for prediction. The optimal number of variables included in the final model was determined by the “tune.block.splsda” command, with 8-fold cross validation repeated 50 times. The nearZeroVar command was applied to the microbiome dataset with the following cut-offs: uniqueCut = 20, freqCut = 15.

## 3 Results

### 3.1 Study design

**FIGURE 1:**
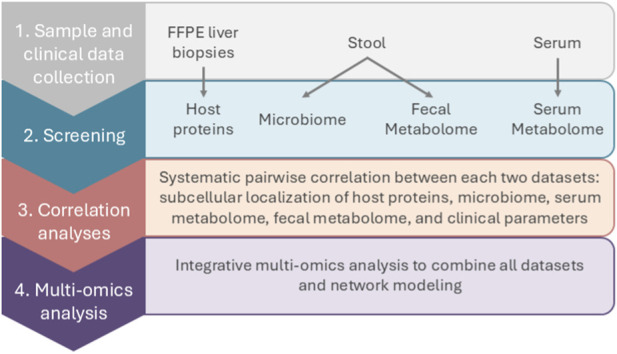
Flowchart showing the approach used to understand the possible associations between SIRT1, FXR, PPARA, and PPARG and the microbiome and metabolome. First, samples (stool, serum, and FFPE from patients, and stool and serum from controls) were collected from MASLD patients and controls. This was followed by IHC, then microbiome and metabolome analysis. Afterward, correlation analysis was employed between dataset pairs. Finally, an integrative analysis between all datasets was performed.

To investigate possible associations between SIRT1, FXR, PPARA, and PPARG and the corresponding microbiome and metabolome, we collected serum and stool samples from patients with MASLD and healthy controls, in addition to FFPE liver biopsies from patients only (Supplementary Tables S1, S2). First, screening of the protein targets in liver biopsies, as well as the microbiome in stool and the metabolome in serum and stool, was performed. Second, pairwise correlation analyses were performed to investigate possible associations between all screened parameters as well as the patients’ clinical data. Finally, an integrative analysis was performed to investigate whether all factors may be correlated (Figure 1).

### 3.2 Screening of host proteins, microbiome, and metabolome

#### 3.2.1 Nuclear and cytoplasmic expression of SIRT1, FXR, PPARA, and PPARG in patients with MASLD/MASH

**FIGURE 2:**
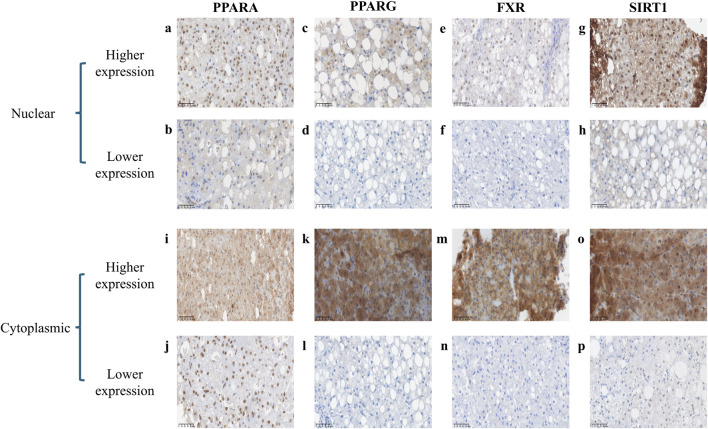
Immunohistochemistry (IHC) of SIRT1, FXR, PPARA, and PPARG. Proteins are stained in brown, while nuclei are stained in blue at ×40 magnification. **(a)** Representative image of the higher nuclear expression of PPARA. **(b)** Representative image of the lower nuclear expression of PPARA. **(c)** Representative image of the higher nuclear expression of PPARG. **(d)** Representative image of the lower nuclear expression of PPARG. **(e)** Representative image of the higher nuclear expression of FXR. **(f)** Representative image of the lower nuclear expression of FXR. **(g)** Representative image of the higher nuclear expression of SIRT1. **(h)** Representative image of the lower nuclear expression of SIRT1. **(i)** Representative image of the higher cytoplasmic expression of PPARA. **(j)** Representative image of the lower cytoplasmic expression of PPARA. **(k)** Representative image of the higher cytoplasmic expression of PPARG. **(l)** Representative image of the lower cytoplasmic expression of PPARG. **(m)** Representative image of the higher cytoplasmic expression of FXR. **(n)** Representative image of the lower cytoplasmic expression of FXR. **(o)** Representative image of the higher cytoplasmic expression of SIRT1. **(p)** Representative image of the lower cytoplasmic expression of SIRT1.

Because the subcellular localization of SIRT1, FXR, PPARA, and PPARG is crucial for their function, we first determined the nuclear and cytoplasmic expression of these protein targets in tissue sections from FFPE tissues of patients with MASLD (Figure 2). We observed different patterns of expression and subcellular localization of SIRT1, FXR, and PPARA, and PPARG among the patients’ FFPE samples and compared them to IHC staining from normal liver tissues obtained from the Human Protein Atlas (www.proteinatlas.org) (Supplementary Figure S1). To explore possible reasons for the observed discrepant expression and subcellular localization, we compared the nuclear and cytoplasmic expression of the protein targets between patients with MASLD and MASH. No significant difference was found, except for a tendency of PPARG to be repressed in patients with MASH (*p-*value = 0.079, Supplementary Figure S2). Our next aim was to investigate the relationship between the host protein targets and the microbiome/metabolome profiles.

#### 3.2.2 Microbiome alteration in patients with MASLD compared to healthy controls

To compare the gut microbiome of patients with MASLD vs. healthy controls, we used 16S amplicon sequencing for taxonomic profiling of the fecal microbiome of 21 patients and 14 healthy controls. Overall, 26 bacterial phyla and 181 genera were identified (Supplementary Tables S3, S4, respectively).

**FIGURE 3:**
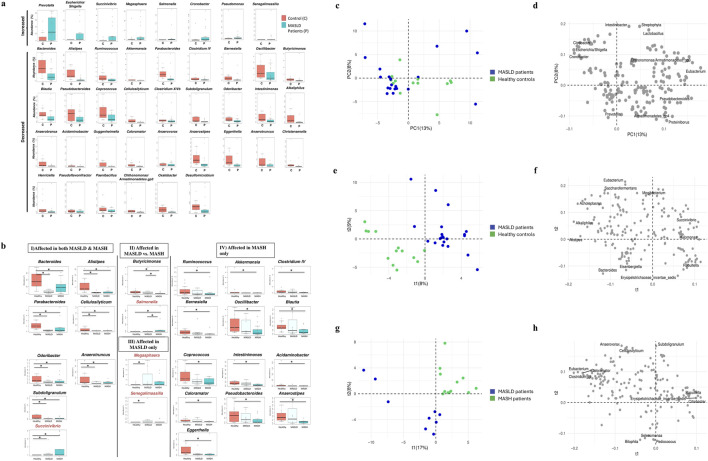
Microbiome alteration in patients with MASLD and MASH compared to healthy controls. **(a)** Boxplots showing the deregulated genera in MASLD patients compared with healthy controls. Genera with increased abundance in patients with MASLD compared to controls are shown in the upper panel. Genera with decreased abundance in patients with MASLD compared to healthy controls are shown in the lower panel. Patients are marked in turquoise, while healthy controls are highlighted in salmon color. The Wilcoxon test was performed. **(b)** Deregulated genera among patients with MASLD, MASH, and healthy controls**.** Panel I shows genera with altered abundance in patients with MASLD and MASH. Panel II shows genera with altered abundance in patients with MASH compared to patients with MASLD. Panel III shows genera with altered abundance in patients with MASLD compared to healthy individuals. Panel IV shows genera with altered abundance in patients with MASH compared to healthy individuals. Patients with MASH are marked in turquoise, patients with MASLD in light blue, and healthy controls are highlighted in salmon. Genera labeled in red are those with increased abundance. The Kruskal–Wallis test was performed using the Benjamini–Hochberg *p*-value adjustment. **(c**, **d)** Microbiome-based PCA score and loading plots of PC1 and PC2 showing separation of patients with MASLD (blue) *versus* healthy controls (green) (*R* ^2^ = 0.508) and contributing components with their assignment, respectively. **(e**, **f)** Score and loading plots of microbiome OPLS showing segregation between patients with MASLD (blue) and healthy controls (green) (R^2^X = 0.23, R^2^Y = 0.928, Q^2^Y = 0.492, pR^2^Y = 0.05, and pQ^2^ = 0.05) and contributing peaks and their assignment, respectively. **(g**, **h)** Score and loading plots of microbiome OPLS showing segregation between patients with MASLD (blue) and patients with MASH (green) (R^2^X = 0.25, R^2^Y = 0.835, Q^2^Y = 0.471, pR^2^Y = 0.45, and pQ^2^ = 0.05) and contributing peaks with their assignment, respectively.

We found 43 genera to be significantly altered in abundance in patients compared to controls (Figure 3a; Supplementary Table S5). Enriched genera in patients include *Prevotella*, *Escherichia/Shigella*, *Succinivibrio*, *Megasphaera*, *Salmonella*, *Cronobacter*, *Pseudomonas*, and *Senegalimassilia*. Conversely, the genera that were less abundant in patients included *Bacteroides*, *Alistipes*, *Ruminococcus*, *Akkermansia*, *Parabacteroides*, *Clostridium IV*, *Barnesiella*, *Oscillibacter*, *Butyricimonas*, *Blautia*, and others (Figure 3a). Our results for *Prevotella*, *Escherichia*/*Shigella*, *Alistipes*, *Oscillibacter*, *Ruminococcus,* and *Coprococcus* are in line with other studies on patients with MASLD (Aron-Wisnewsky et al., 2020; Li et al., 2021). *Alistipes* abundance was low in our cohort. *Alistipes* was reported to be protective and anti-inflammatory, and its decrease is accompanied by advancement to fibrosis (Parker et al., 2020). Contradictory results for *Prevotella* and *Bacteroides* are reported (Aron-Wisnewsky et al., 2020). This discrepancy might be attributed to dietary habits, where *Prevotella* was associated with dietary carbohydrates, while *Bacteroides* was associated with dietary protein and animal fat (Wu et al., 2011; Dahl et al., 2020). Additionally, individuals with a high *Prevotella*/*Bacteroides* ratio were reportedly susceptible to regaining weight, which is associated with low dietary fiber intake and impaired glucose metabolism (Hjorth et al., 2020). Although the abundance of 43 bacterial genera was significantly altered in our cohort, microbial evenness, richness, and diversity (as measured by the Shannon index) did not differ between patients and healthy controls (Supplementary Figure S3). This aligns with two other studies (Boursier et al., 2016; Rodriguez-Diaz et al., 2022), yet contradicts the study by Yang L. et al. (2022).

We further segregated the patients into patients with MASLD and patients with MASH and found 36 genera to be altered (Supplementary Tables S6, S7). In both patients with MASLD and patients with MASH compared to healthy controls, *Bacteroides*, *Alistipes*, *Parabacteroides*, *Cellulosilyticum*, *Odoribacter*, *Anaerotruncus*, and *Subdoligranulum* were depleted, whereas *Succinivibrio* was enriched (Figure 3b). *Megasphaera* and *Senegalimassilia* were augmented in patients with MASLD, while 13 genera were depleted only in patients with MASH compared to healthy controls: *Ruminococcus*, *Akkermansia*, *Clostridium IV*, *Barnesiella*, *Oscillibacter*, *Blautia*, *Coprococcus*, *Intestinimonas*, *Acidaminobacter*, *Caloramator*, *Pseudobacteroides*, *Anaerostipes*, *and Eggerthella* (Figure 3b). Finally, *Butyricimonas* was reduced, while *Salmonella* was enriched in patients with MASH compared to MASLD (Figure 3b). One *Butyricimonas* species was recently found to avert HFD-induced diabetes and metabolic disorders in mice (Lee et al., 2022). In contrast, *Salmonella* was found enriched in MASLD in a population-based study (Patel et al., 2024).

We further performed principal component analysis (PCA) and orthogonal partial least square-discriminant analysis (OPLS-DA) to reduce the dimensionality of the data and determine whether the microbiome could differentiate between MASLD patients and healthy controls and which genera contributed to the difference. The PCA score plot showed mild separation between patients with MASLD and controls, where the first two components explained 21% of the total variation (R^2^X = 0.508 with seven components) (Figure 3c; Supplementary Table S8). Top contributing variables for this separation were *Eubacterium*, *Pseudobacteroides*, *Chthonomonas/Armatimonadetes gp3*, *Escherichia/Shigella*, *Cronobacter*, *Citrobacter*, *Lactobacillus*, *Intestinibacter*, *Streptophyta*, *Prevotella*, *Armatimonadetes gp4*, and *Proteiniborus* (Figure 3d; Supplementary Table S9). Furthermore, OPLS suggested the following genera to be contributing to the segregation between patients and healthy controls: *Raoultella*, *Halomonas*, *Succinivibrio*, *Alkaliphilus*, *Acholeplasma*, *Alistipes*, *Eubacterium*, *Saccharofermentans*, *Mogibacterium*, *Erysipelotrichaceae* *incertae sedis*, *Bacteroides*, and *Eisenbergiella* (R^2^Y = 0.928, Q^2^Y = 0.492, pR^2^Y = 0.05, and pQ^2^ = 0.05) (Figures 3e,f; Supplementary Tables S10, S11). The OPLS model explains 92.8% of the response (MASLD vs. healthy controls) with nearly moderate and statistically significant predictive power (Figures 3e,f). Both PCA and OPLS exhibited that *Eubacterium* was among the contributing genera for the segregation between patients and controls.

We performed another OPLS analysis for the microbiome between patients with MASLD and MASH (Figure 3g; Supplementary Table S12). OPLS analysis depicted the following to be contributing to the difference between patients with MASLD and those with MASH: *Citrobacter*, *Raoultella*, *Erysipelotrichaceae incertae sedis*, *Clostridium III*, *Eubacterium*, *Caloramator*, *Anaerovorax*, *Subdoligranulum*, *Cellulosilyticum*, *Pediococcus*, *Bilophila*, and *Selenomonas* (R^2^Y = 0.835, Q^2^Y = 0.471, pR^2^Y = 0.45, and pQ^2^ = 0.05) (Figure 3h; Supplementary Table S13). Again, *Eubacterium* was found to be a variable responsible for segregation. Although this OPLS model explains 83.5% of the response with a statistically significant predictive power (Q^2^), it has an insignificant pR^2^ value, showing that data might be overfitted. Nevertheless, it gives an indication of the contributing factors.

#### 3.2.3 Metabolome alteration in patients with MASLD compared to healthy controls

The gut microbiome can affect host targets through released metabolites (Boccuto et al., 2023); accordingly, we investigated the metabolic profiles of patients with MASLD and healthy controls. We analyzed both fecal and serum metabolites to assess the direct and indirect impact of the microbiome, respectively, and detected 185 and 169 compounds in fecal and serum samples, respectively (Supplementary Tables S14, S15), respectively. Representative GC/MS chromatograms for peaks are shown in Supplementary Figure S4.

**FIGURE 4:**
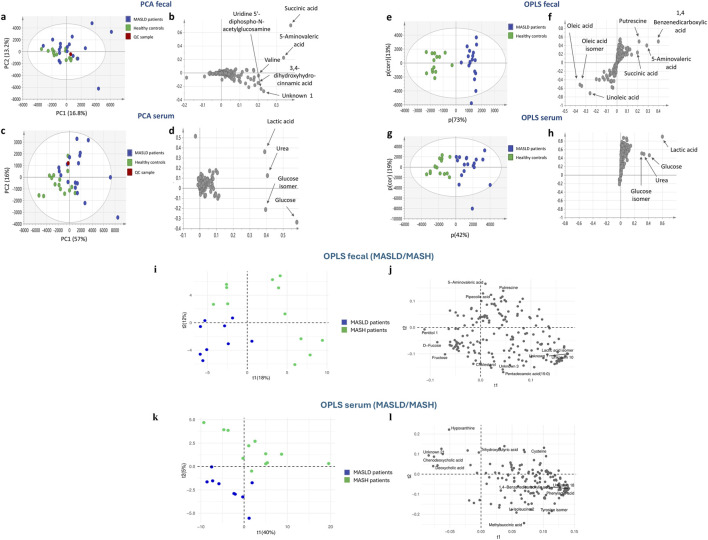
Metabolome alteration in patients with MASLD and MASH compared to healthy controls. **(a**, **b)** Fecal metabolome-based PCA score and loading plots of PC1 and PC2 showing separation of patients with MASLD *versus* healthy controls and showing the most discriminatory fecal metabolites and their assignment, respectively. **(c**, **d)** Serum metabolome-based PCA score and loading plots of PC1 and PC2 showing separation of patients with MASLD *versus* healthy controls and showing the most discriminatory serum metabolites and their assignment, respectively. Patients with MASLD are marked in blue, and healthy controls are marked in green. The quality control (QC) sample is shown in red. **(e**, **f)** Fecal metabolome-based OPLS score and S-plots showing segregation between patients with MASLD and healthy controls (*R* ^2^ cum = 0.86, Q^2^ cum = 0.66, *p*-value = 1.9e−006) and displaying contributing peaks with their assignment, respectively. **(g**, **h)** Serum metabolome-based OPLS score and S-plots showing segregation between patients with MASLD and healthy controls (*R* ^2^ cum = 0.62, Q^2^ cum = 0.50, *p*-value = 0.002), and depicting contributing peaks with their assignment, respectively. MASLD patients are marked in blue, and healthy controls are marked in green. **(i**, **j)** Fecal metabolome-based OPLS score and loading plots showing segregation between patients with MASLD and MASH (R^2^X = 0.308, R^2^Y = 0.673, Q^2^Y = 0.273, pR^2^Y = 1.05, and pQ^2^ = 0.05), and fecal metabolites contributing peaks and their assignment, respectively. **(k**, **l)** Serum metabolome-based OPLS score and loading plots showing segregation between patients with MASLD and MASH (R^2^X = 0.445, R^2^Y = 0.848, Q^2^Y = 0.268, pR^2^Y = 0.05, and pQ^2^ = 0.2) and contributing peaks and their assignment, respectively. Patients with MASLD are marked in blue, while patients with MASH are shown in green.

To assess differences between the metabolomes of patients with MASLD and healthy controls, we analyzed the data with PCA and OPLS. The PCA score plot for the fecal metabolome showed clear separation between patients and controls, albeit with low variance coverage along the first two components (30% of the total variance, Figure 4a). The loading plot showed the following to be discriminatory between patients compared to healthy controls: 5-aminovaleric acid (5-aminopentanoic acid), succinic acid, valine, 3,4-dihydroxyhydrocinnamic acid, and uridine 5′-diphospho-N-acetylglucosamine (Figure 4b). In contrast, the PCA score plot for the serum metabolome showed higher separation between patients and controls, explaining more variation, as the first two components explained 73% of the total variance (Figure 4c). The corresponding loading plot depicted lactic acid, urea, and glucose to be discriminatory between patients and controls (Figure 4d).

We used OPLS to further validate PCA results and improve separation between groups. The OPLS score plot of fecal metabolites of patients compared to controls validated the PCA results, displaying further separation (*R* ^2^ cum = 0.86, Q^2^ cum = 0.66, *p*-value = 1.9e−006, Figure 4e), and more metabolites mediating the variation (Figure 4f). Putrescine, 1,4-benzenedicarboxylic acid, succinic acid, and 5-aminovaleric acid appear to be positively correlated with patients with MASLD and negatively correlated with oleic acid and its isomer, and linoleic acid, which were also negatively correlated with MASLD status (Figure 4f). Similarly, OPLS applied to serum metabolites further reinforced the PCA results (Figure 4g) and showed the same markers responsible for sample segregation that are positively correlated with MASLD (*R* ^2^ cum = 0.62, Q^2^ cum = 0.50, *p*-value = 0.002, Figure 4h). To avoid the masking effect of major metabolites, we reperformed OPLS after the removal of sugars, the major peaks detected by GC/MS. Interestingly, OPLS showed better distinct separation between patients with MASLD and healthy controls (*R* ^2^ cum = 0.73, Q^2^ cum = 0.56, *p*-value = 0.003, Supplementary Figure S5).

We expanded the same analysis to understand the differences in the metabolome between patients with MASLD and MASH (Figures 4i–l). Fecal metabolites found to be more associated with patients with MASH were 5-aminovaleric acid, putrescine, pipecolic acid, and lactic acid, whereas fructose, D-fucose, and pentitol were more associated with patients with MASLD (R^2^Y = 0.673, Q^2^Y = 0.273, pR^2^Y = 1.05, and pQ^2^ = 0.05, Figures 4i,j) (Supplementary Tables S16, S17). The serum metabolites found responsible for the segregation were hypoxanthine, trihydroxybutyric acid, and cysteine, which were more associated with patients with MASH, while deoxycholic, chenodeoxycholic, and methylsuccinic acids were more associated with patients with MASLD (R^2^Y = 0.848, Q^2^Y = 0.268, pR^2^Y = 0.05, and pQ^2^ = 0.2, Figures 4k,l) (Supplementary Tables S18, S19). OPLS models for MASLD/MASH did not show strong prediction power (Q^2^), suggesting the need for further validation with a larger sample size.

Our models prominently suggested 5-aminovaleric acid as a discriminatory metabolite. To our knowledge, there are no reports about 5-aminovaleric acid. Nevertheless, its derivative N,N,N-trimethyl-5-aminovaleric acid (TMAVA), which is a gut microbiome metabolite, exacerbates fatty liver through diminishing carnitine synthesis (Zhao et al., 2020). Fecal succinic acid was similarly predicted by our models to be discriminatory. Succinic acid has a profibrogenic role, activating hepatic stellate cells and promoting MASLD progression to fibrosis and cirrhosis (Yang and Zhang, 2021). We also show fecal putrescine to be associated with MASLD or MASH. Hepatic putrescine was found to be elevated in MASLD and positively correlated with SAF score (steatosis, activity, fibrosis score) (Nunez-Sanchez et al., 2024). Furthermore, fecal putrescine was found to be related to colorectal cancer-associated microbes (Yang et al., 2019).

We also observed serum lactic acid, urea, and glucose to be discriminatory between patients and controls. Lactic acid was also found to be dysregulated in patients with MASLD (Gottlieb et al., 2021). A Chinese study reported that lactic acid levels were associated with increased risk of MASLD in type 2 diabetes mellitus patients (Ma et al., 2023). Increased serum urea in MASLD patients was also reported (Sun et al., 2024). Urea synthesis is a special liver function that is jeopardized in MASLD, leading to ammonia accumulation that is suspected to contribute to inflammation, fibrogenesis, and a global negative impact on other organs (Thomsen et al., 2023). Furthermore, our glucose results are especially interesting, as the triglyceride-glucose (TyG) index has recently been considered a measure of insulin resistance and could be potentially used with other parameters for early screening of MASLD (Xue Y. et al., 2022).

##### 3.2.3.1 Functional classification of altered metabolites

**FIGURE 5:**
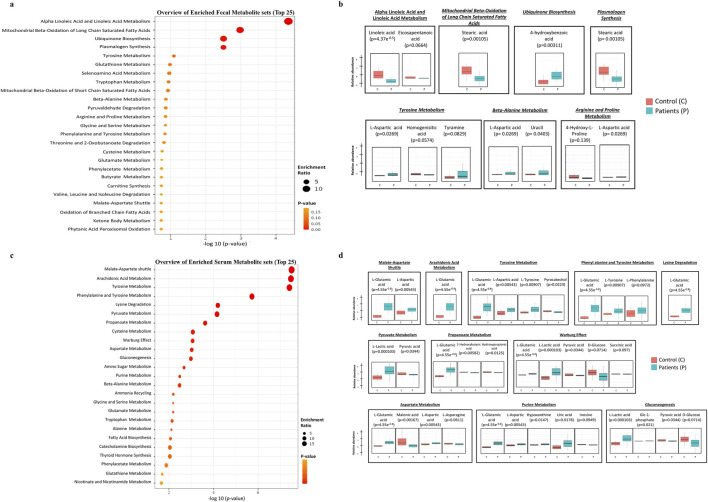
Enrichment analysis of fecal and serum metabolomes of patients with MASLD compared to healthy controls. **(a)** Results of enrichment analysis showing the top 25 affected metabolic pathways in the fecal metabolome of patients with MASLD vs. healthy controls. **(b)** Boxplots showing the top metabolic pathways with the most prominent fecal metabolites that are most significantly altered in patients with MASLD vs. healthy controls. **(c)** Results of enrichment analysis showing the top 25 affected metabolic pathways in the serum metabolomes of patients with MASLD vs. healthy controls. **(d)** Boxplots showing the top metabolic pathways with their most prominent serum metabolites that are most significantly altered in patients with MASLD vs. healthy controls. Circles in red, with the largest diameter, show metabolic pathways with the highest enrichment ratios and the most significant *p*-value in patients with MASLD vs. healthy controls. Turquoise bars represent patients, and orange bars represent controls.

To assess the functional impact of altered metabolites depicted in the metabolomes of patients with MASLD, we performed pathway enrichment analysis using MetaboAnalyst 5.0. Pathway enrichment analysis of the fecal metabolome showed several pathways significantly affected with a high enrichment ratio in patients with MASLD (Figures 5a,b; Supplementary Figure S6). Altered fecal metabolites included linoleic and eicosapentaenoic acids in the α-linoleic acid and linoleic acid metabolic pathways, stearic acid in the mitochondrial beta-oxidation of long-chain saturated fatty acids metabolic pathway and plasmalogen synthesis pathway, and 4-hydroxybenzoic acid in the ubiquinone biosynthesis pathway (Figure 5b; Supplementary Figure S6). In addition, there was also enriched L-aspartic acid and tyramine in the tyrosine synthesis pathway. L-aspartic acid was enriched in the beta-alanine metabolism pathway with uracil. Moreover, L-aspartic acid was enriched in the arginine and proline metabolism pathway and the beta-alanine metabolism pathway (Supplementary Figure S6).

In contrast, pathway enrichment analysis for the serum metabolome revealed more altered and different pathways than those from the fecal metabolome. The most significantly altered pathways were the malate-aspartate shuttle pathway, the arachidonic acid metabolism pathway, and tyrosine metabolism. The next pathways significantly affected were amino acid metabolism, pyruvate metabolism, propanoate metabolism, the Warburg effect, aspartate metabolism, and gluconeogenesis (Figures 5c,d; Supplementary Figure S7). The common serum metabolite enriched in all pathways except gluconeogenesis and the pyruvate metabolism pathway was L-glutamic acid (Figure 5d; Supplementary Figure S7). In addition, L-lactic acid, which was found to be discriminatory by our PCA and OPLS analyses, was also enriched in patients with MASLD compared to healthy controls. L-lactic acid was enriched in pyruvate metabolism, the Warburg effect, and gluconeogenesis pathways (Figure 5d; Supplementary Figure S7). Recently, lactic acid was shown to be oncogenic (Lv et al., 2023).

It would be interesting to investigate whether increased lactic acid might explain the extrahepatic malignancies exhibited by patients with MASLD, especially because the Warburg pathway, a hallmark for cancer cells (Schwartz et al., 2017), was enriched in serum metabolites. Moreover, serum L-aspartic acid was enriched in patients with MASLD (Figure 5d; Supplementary Figure S7), similar to fecal L-aspartic acid (Figure 5b; Supplementary Figure S6). In serum, L-aspartic acid was enriched in the malate-aspartate shuttle pathway, tyrosine metabolism, aspartate metabolism, and purine metabolism pathways (Figure 5d; Supplementary Figure S7). L-tyrosine was enriched in patients with MASLD in the phenylalanine and tyrosine metabolism pathway, while hypoxanthine and uric acid were enriched in purine metabolism pathways (Figure 5d; Supplementary Figure S7).

### 3.3 Correlation analyses between dataset pairs

#### 3.3.1 Microbiome–metabolome correlation analysis

**FIGURE 6:**
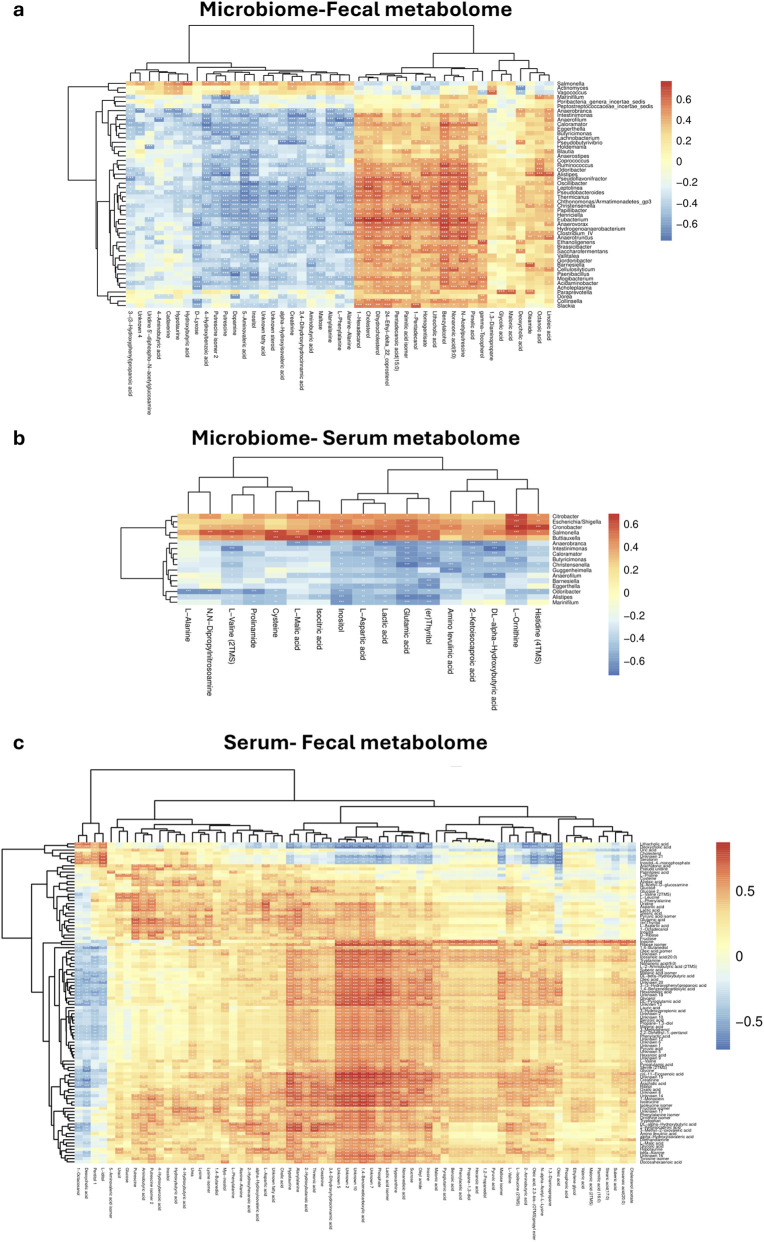
Associations between the microbiome–metabolome and the fecal–serum metabolome. **(a)** Heatmap showing correlations between microbial genera and fecal metabolites. **(b)** Heatmap showing correlations between microbial genera and serum metabolites. **(c)** Heatmap showing correlations between fecal metabolomes (horizontal) and serum metabolomes (vertical). Spearman correlation analysis was employed using the pairwise method. All heatmaps show a Spearman correlation coefficient R cutoff of ±0.6 and their corresponding statistically significant *p*-values. A *p*-value less than 0.05 was considered statistically significant. *** *p* < 0.001, ** *p* < 0.01, * *p* < 0.05. Holm adjustment for multiple comparisons was performed.

We investigated the microbiome–metabolome associations by performing correlation analysis between bacterial genera and metabolites (Figures 6a,b, respectively). Spearman correlation analysis was also performed between microbial phyla or genera against classes or compounds of fecal or serum metabolites (Supplementary Figures S8, S9; Supplementary Tables 20–35). A specific interest in such correlation studies is to determine patterns of co-abundance between microbial genera and fecal or serum metabolites. Our data appeared to segregate into two main clusters: one with positive correlations and the other with negative correlations. (Supplementary Tables S20, S21).

##### 3.3.1.1 Microbiome-fecal metabolome correlation analysis

With regards to fecal metabolites, strong positive correlations (with *r* > 0.7) were observed for benzyl alcohol with *Anaerovorax*, *Ruminococcus*, *Hydrogenoanaerobacterium*, and *Eubacterium*. The second most correlated fecal metabolite is N-acetylputrescine with *Eubacterium* and *Anaerotruncus*, as well as cholesterol with *Eubacterium* and *Leptolinea*. Moreover, dihydrocholesterol and 1-hexadecanol were positively correlated with *Eubacterium*. On the other hand, 5-aminovaleric acid had the most negative correlations (*r* < −0.7) with *Alistipes*, *Oscillibacter*, *Pseudobacteroides*, and *Thermicanus*. Inositol also had negative associations (*r* < −0.7) with *Oscillibacter* and *Anaerotruncus*, as well as putrescine with *Alistipes* and *Marinifilum*. Moreover, L-phenylalanine was negatively correlated with *Anaerobium*, creatinine with *Pseudobutyrivibrio*, dopamine with *Paenibacillus*, and 4-hydroxybenzoic acid with *Caloramator* (Figure 6a).

##### 3.3.1.2 Microbiome-serum metabolome correlation analysis

Correlations between microbial genera and serum metabolites mostly involved amino acids. For example, glutamic acid was negatively correlated with *Christensenella*, *Alistipes*, *Intestinimonas*, *Caloramator*, *and Butyricimonas*. L-valine was negatively correlated with *Intestinimonas*, L-alanine with *Odoribacter*, and aminolevulinic acid with *Guggenheimella*. On the other hand, L-ornithine was positively correlated with *Cronobacter*, *Citrobacter*, *and Escherichia/Shigella*. Histidine was positively correlated with *Cronobacter*. Additionally, aspartic acid had a positive correlation with *Salmonella*, and cysteine had a positive correlation with *Buttiauxella* and *Salmonella*. There were also negative correlations between α-hydroxybutyric acid and *Intestinimonas* and *Anaerofilum*. Next was lactic acid, with a negative correlation with *Anaerobranca* and a positive correlation with *Salmonella*. Sugar alcohols depicted several correlations, mainly with inositol and (er)thyritol. (Er)thyritol showed negative associations with *Alistipes*, *Eggerthella*, *Butyricimonas*, and *Barnesiella*. Meanwhile, inositol had a negative association with *Marinifilum* and *Alistipes* (Figure 6b; Supplementary Tables S22, S23).

We also investigated co-associations between fecal and serum metabolomes to explore common metabolites. Correlation analysis yielded a high number of co-associations (Supplementary Tables S36, S37) (Figure 6c).

#### 3.3.2 Associations of microbiome and fecal/serum metabolome with SIRT1, FXR, PPARA, and PPARG

The main goal of this study was to assess whether nuclear or cytoplasmic expression of SIRT1, FXR, PPARA, and PPARG is affected by either microbiome or metabolome. Accordingly, we investigated possible pairwise associations among the subcellular localization of each protein and each dataset.

##### 3.3.2.1 Host protein–microbiome correlation analysis

**FIGURE 7:**
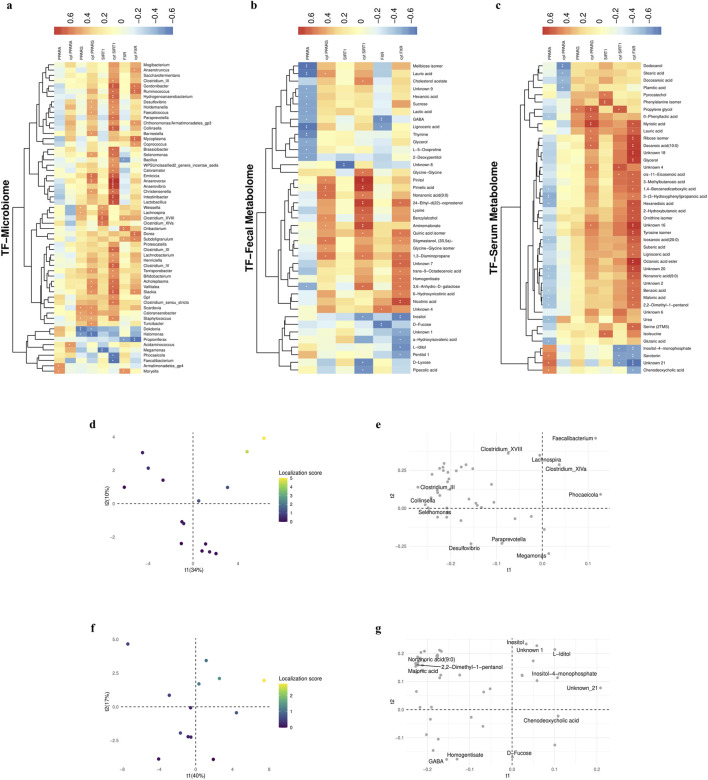
Correlation analysis between SIRT1, FXR, PPARA, and PPARG and microbiome/metabolome in patients with MASLD. **(a)** Heatmap showing the correlation between SIRT1, FXR, PPARA, and PPARG (nuclear and cytoplasmic expression) and the microbial genera of patients with MASLD. **(b)** Heatmap showing the correlation between SIRT1, FXR, PPARA, and PPARG (nuclear and cytoplasmic expression) and fecal metabolites in patients with MASLD. **(c)** Heatmap showing the correlation between SIRT1, FXR, PPARA, and PPARG (nuclear and cytoplasmic expression) and serum metabolites in patients with MASLD. Spearman correlation analysis was employed using the pairwise method, and heatmaps show a Spearman correlation coefficient R cutoff of ±0.5 and their corresponding statistically significant *p*-value. A *p*-value less than 0.05 was considered statistically significant. *** *p* < 0.001, ** *p* < 0.01, * *p* < 0.05. Holm adjustment for multiple comparisons was applied. **(d)** Score plot of the PLS model between the SIRT1 localization score and correlated microbial genera of patients with MASLD (R^2^X = 0.576, R^2^Y = 0.77, Q^2^ = 0.392, pR^2^Y = 0.35, and pQ^2^ = 0.05). **(e)** Loading plot derived from the PLS model of the SIRT1 localization score and its correlated microbial genera of patients with MASLD, showing contributing peaks and their assignment. **(f)** Score plot of the PLS model between the FXR localization score and correlated fecal and serum metabolites of patients with MASLD (R^2^Y = 0.772, Q^2^ = 0.446, pR^2^Y = 0.4, and pQ^2^ = 0.05). **(g)** Loading plot derived from the PLS model of the FXR localization score and its correlated fecal and serum metabolites of patients with MASLD, showing contributing peaks and their assignment.

Regarding the microbiome, the strongest and highest number of correlations was with cytoplasmic SIRT1 (cytSIRT1), which was positively correlated with *Anaerovorax*, *Intestinibacter*, and *Anaerovibrio*, *Lactobacillus*, *Gordonibacter*, *Emticicia*, *Collinsella*, *Clostridium IV*, *Slackia*, *Hydrogenoanaerobacterium*, *Clostridium XI*, *Brassicibacter*, *Vallitalea*, *Christensenella*, *Acholeplasma*, *Paraprevotella*, *Ruminococcus*, *Selenomonas*, and *Clostridium sensu stricto*. Nuclear SIRT1 (SIRT1) was positively correlated with *Clostridium XVIII*, and cytoplasmic FXR (cytFXR) was positively correlated with *Ruminococcus*, *Dorea*, *Mycoplasma*, and *Gordonibacter* yet negatively correlated with *Propioniferax*. Cytoplasmic PPARG (cytPPARG) was positively correlated with *Emticicia* (Figure 7a; Supplementary Tables S38, S39).

##### 3.3.2.2 Host protein–metabolome correlation analysis

With regards to the fecal metabolome, again cytSIRT1 showed more and stronger associations with pinitol, pimelic acid, 24-ethyl-δ(22)-coprostenol, aminomalonate, and lysine. Moreover, cytFXR was positively correlated with nicotinic acid but negatively correlated with inositol. Nuclear FXR was negatively correlated with fucose. Nuclear PPARA had mostly negative correlations with lauric acid, melibiose isomer, lignoceric acid, and thymine (Figure 7b; Supplementary Tables S40, S41).

Regarding the serum metabolome, the strongest associations were observed with cytFXR, which showed positive correlations with octanoic acid ester, decanoic acid, hexanedioic acid, glycerol, tyrosine isomer, and ribose isomer and negative correlations with serotonin and inositol-4-monophosphate. Similar to cytFXR, cytSIRT1 also showed negative correlations with serotonin and inositol-4-monophosphate. In addition, cytPPARG displayed a positive correlation with myristic acid, while cytPPARA showed a negative correlation with dodecanol. Nuclear PPARA also showed most correlations with negative co-associations with glutaric acid, hexanedioic acid, 3-(3-hydroxyphenyl) propanoic acid, and urea. Nuclear PPARG depicted a positive correlation with propylene glycol, while nuclear SIRT1 exhibited positive correlations with isoleucine, phenylalanine isomer, and pyrocatechol. Nuclear FXR showed no associations with serum metabolites (Figure 7c; Supplementary Tables S42, S43).

##### 3.3.2.3 Can the correlated microbiome or metabolome predict the localization of host proteins?

We next tested whether the correlated microbiome or metabolome can predict nuclear localization of our targets. Accordingly, a localization score was calculated for each patient, whereby the nuclear score was divided by the cytoplasmic score. This localization score was further used to build a partial least squares (PLS) model between the localization scores of SIRT1, FXR, PPARA, and PPARG, and the significantly correlated microbiome and metabolome.

Nuclear localization of PPARA and PPARG could not be predicted; however, the model for SIRT1 subcellular localization, using the correlated microbial genera, was promising and could explain 77% (R^2^Y) of the variance in localization score with three components, and a moderate predictive power (Q^2^) (R^2^Y = 0.77, Q^2^ = 0.392, pR^2^Y = 0.35, and pQ^2^ = 0.05, Figure 7d). *Faecalibacterium*, *Clostridium XlVa*, and *Phocaeicola* were more associated with more nuclear SIRT1 expression and thus higher localization scores (Figure 7e; Supplementary Figure S10). On the other hand, *Paraprevotella*, *Desulfovibrio*, *Clostridium III*, *Collinsella,* and *Selenomonas* were more associated with cytoplasmic SIRT1 expression and thus lower localization scores (Figure 7e; Supplementary Figure S10; Supplementary Tables S44, S45).

Similarly, FXR had a promising PLS model, in which microbial genera and fecal and serum metabolites correlated with nuclear or cytoplasmic FXR (R^2^Y = 0.845, Q^2^ = 0.519, pR^2^ = 0.55, and pQ^2^ = 0.1). To have a better pQ^2^, we repeated the model with the correlated fecal and serum metabolome only, and this model explained 77% (R^2^Y) of the response (localization score of FXR) with two components and a moderate predictive power (Q^2^) (R^2^Y = 0.772, Q^2^ = 0.446, pR^2^Y = 0.4, and pQ = 0.05) (Figure 7f; Supplementary Figure S11). Our model suggests that fecal iditol, as well as serum inositol-4-monophosphate, are associated with higher localization scores and thus more nuclear FXR expression (Figure 7g). On the other hand, fecal GABA and homogentisate, as well as serum nonanoic and malonic acids and 2,2-dimethyl-1-pentanol, are associated with lower localization score and thus cytoplasmic FXR expression (Figure 7g; Supplementary Tables S46, S47).

#### 3.3.3 Clinical associations with SIRT1, FXR, PPARA, and PPARG expression, microbiome, and fecal/serum metabolome

**FIGURE 8:**
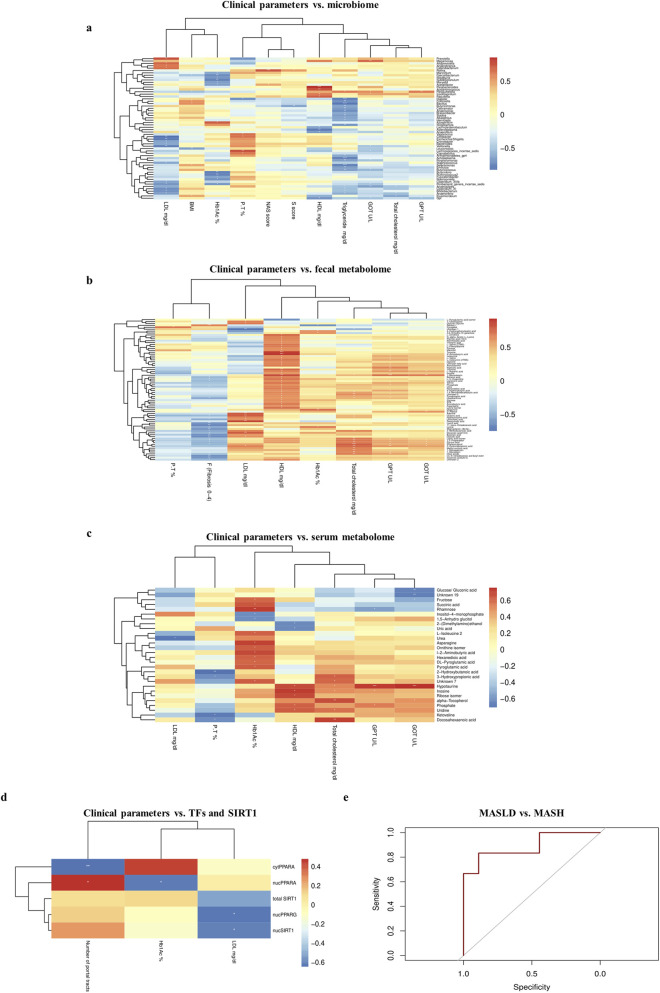
Clinical associations with microbiome, fecal/serum metabolome, and SIRT1, FXR, PPARA, and PPARG. **(a)** Heatmap showing correlations between clinical parameters and microbiome in patients with MASLD. **(b)** Heatmap showing correlations between clinical parameters and fecal metabolome in patients with MASLD. **(c)** Heatmap showing correlations between clinical parameters and serum metabolome in patients with MASLD. **(d)** Heatmap showing correlations between clinical parameters and SIRT1, FXR, PPARA, and PPARG in patients with MASLD. Spearman correlation analysis was employed using the pairwise method; heatmaps show a Spearman correlation coefficient R cutoff of ±0.6 and their corresponding statistically significant *p*-value. A *p*-value less than 0.05 was considered statistically significant. *** *p* < 0.001, ** *p* < 0.01, * *p* < 0.05. Holm adjustment for multiple comparisons was performed. **(e)** ROC AUC curve for logistic regression model of the three microbial genera classifying patients with MASLD vs. patients with MASH (*p*-value = 0.002).

We further investigated whether SIRT1, FXR, PPARA, and PPARG expression, microbiome, and fecal/serum metabolomes were correlated with the clinical parameters of the patients (Figure 8; Supplementary Tables S48–55). Clinical parameters showed multiple associations with microbiome and fecal/serum metabolome (Figure 8). Interestingly, clinical parameters also showed a few correlations with our targets, more specifically, PPARs (Figure 8).

##### 3.3.3.1 Clinical data–microbiome correlation analysis

Most co-associations with the microbiome were with triglyceride levels in serum and were negative. LDL and Hb1Ac% were also negatively correlated with several features (Figure 8a). On the other hand, positive correlations were identified with high-density lipoprotein (HDL), prothrombin time (P.T%), and low-density lipoprotein (LDL) (Figure 8a). Interestingly, the NAS that differentiates between MASLD and MASH is only correlated with the microbiome (Figure 8a). The NAS grades the histopathological assessment of liver damage on a scale of 0–8, according to the degree of assessed steatosis, inflammation, and ballooning. Of note, every clinical parameter was mostly associated with different sets of microbial genera. Whether these are specific to each genus needs further investigation.

To date, the most accurate differentiation between MASLD and MASH is histopathological through the use of the NAS (Piazzolla and Mangia, 2020): a NAS ≥5 indicates MASH, while a NAS <5 indicates MASLD (Kleiner et al., 2005). In our data, the NAS only correlated with the microbiome; therefore, we investigated whether a logistic regression model that uses those microbial genera can classify MASLD/MASH patients. We tested microbial genera that were correlated with the NAS (r > 0.4) and statistically significant. Our logistic regression model suggested that *Rothia*, *Acetatifactor*, and *Haemophilus* could possibly differentiate between patients with MASLD and patients with MASH with an ROC AUC (receiver-operator characteristic area under the curve) = 0.89 and *p*-value = 0.002 (Figure 8e).

##### 3.3.3.2 Clinical data–metabolome correlation analysis

Regarding the metabolome, fecal metabolites were mostly correlated with HDL, followed by total cholesterol, LDL, and GPT (Figure 8b), all of which were positive correlations. Most negative correlations with fecal metabolites were with fibrosis score (including lactic acid isomer and lauric acid, Figure 8b). Finally, serum metabolites showed the fewest correlations: some were positive, for example, with Hb1Ac%, followed by HDL and total cholesterol (Figure 8c), while others were negative, for example, with P.T% (Figure 8c).

##### 3.3.3.3 Clinical data–host proteins correlation analysis

Concerning the subcellular localization of the liver proteins, nuclear PPARA was positively correlated with the number of portal tracts in FFPE samples of patients with MASLD, while the cytoplasmic PPARA was negatively correlated (Figure 8d). Nuclear PPARA also showed a negative correlation with HbA1c%. Moreover, LDL had negative correlations with nuclear PPARG and SIRT1 (Figure 8d).

### 3.4 Multi-omics analysis

MASLD is multifactorial and complex, and as our results delineated multiple correlations between pairs of datasets, we sought to explore how all five datasets (microbiome, fecal metabolome, serum metabolome, the protein targets, and clinical data) correlate, integrate, and whether they can differentiate between MASLD and MASH. Accordingly, the DIABLO (data integration analysis for biomarker discovery using latent variable approaches for omics studies) analysis tool was applied. DIABLO analysis integrates multiple datasets to examine their correlations and how they help explain a categorical outcome, in our case, MASLD vs. MASH.

**FIGURE 9:**
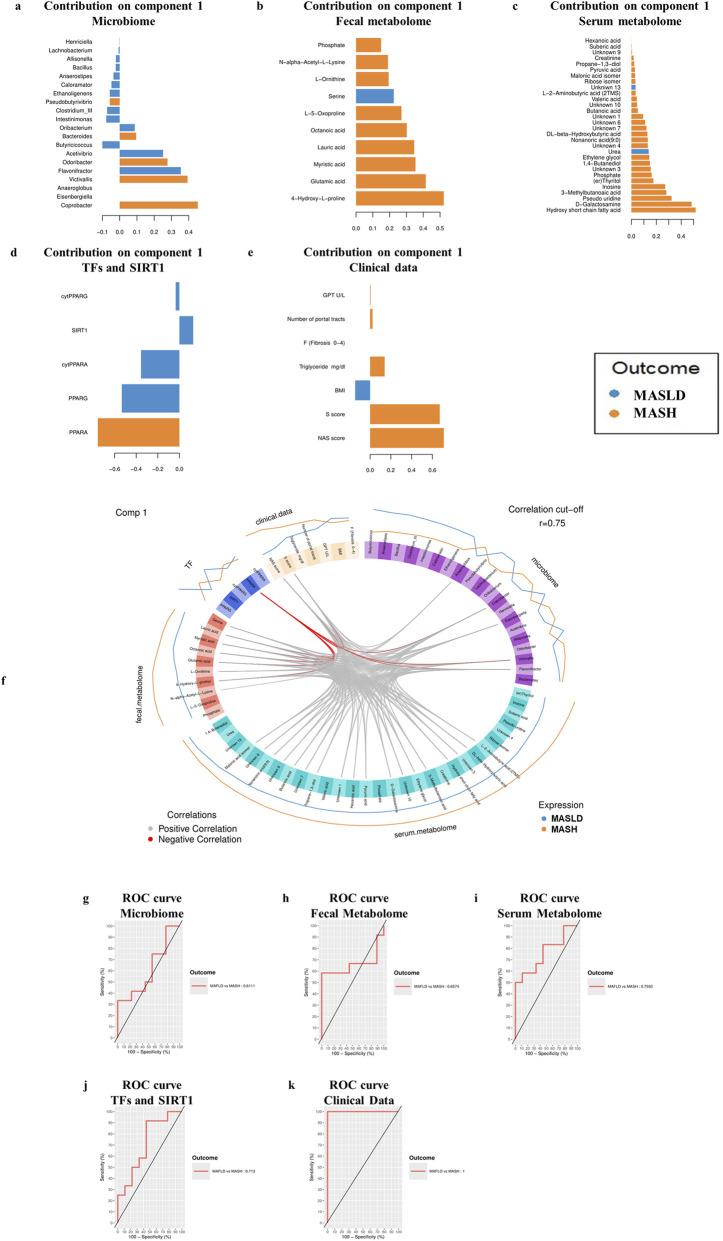
DIABLO analysis showing multi-omics signature of patients with MASLD. **(a–e)** Loading plots for variables selected on component 1 suggested by DIABLO analysis in each dataset. The length of the bars corresponds to the value of their coefficient, whether positive or negative. Variables are ordered from bottom to top according to their contribution. Colors indicate the class: MASLD (blue) or MASH (orange) for which the median expression level is the highest for each variable. **(f)** Circos plot showing selected variables from DIABLO analysis and their correlations. The plot shows correlations exceeding 0.75 between the selected variables. The outer lines demonstrate the expression levels of selected variables in each group: MASLD (blue) or MASH (orange). The inner lines show correlations between variables, with positive correlations in grey and negative correlations in red. Lines showing average expression levels of selected variables illustrate that these variables can discriminate between MASLD and MASH patients. **(g–k)** Validation of the DIABLO analysis with ROC AUC. **(g)** Microbiome ROC AUC = 61% (*p* = 0.394). **(h)** Fecal metabolome ROC AUC = 66% (*p* = 0.227). **(i)** Serum metabolome ROC AUC = 76% (*p* = 0.0466). **(j)** TFs and SIRT1 ROC AUC = 71% (*p* = 0.102). **(k)** Clinical data ROC AUC = 100% (*p* = 0.0001).

We computed and implemented pairwise correlations between pairs of datasets, finding strong correlations (Supplementary Table S56). This analysis suggested one component, for which 6,048 models were fitted with nrepeat (50) to yield suggested variables for each dataset that might contribute to the model (Figures 9a–e; Supplementary Table S57). Because datasets were highly correlated, a weight of 0.5 was used for the design matrix. A circos plot illustrating correlations (*r* > 0.75) between the selected variables from each dataset showed most negative correlations between PPARA with elements from the microbiome and fecal metabolome (Figure 9f). On the other hand, most positive correlations are between NAS values and steatosis grade (S score) with elements from the microbiome and the fecal and serum metabolomes. Our model indicates selected features that could potentially discriminate between patients with MASLD and MASH. ROC AUC analysis was calculated to validate our model (Figures 9g–k). The predictive power of our model is driven by the microbiome (AUC = 61%, Figure 9g), fecal metabolome (AUC = 66%, Figure 9h), serum metabolome (AUC = 76%, Figure 9i), protein targets (AUC = 71%, Figure 9j), and clinical data (AUC = 100%, Figure 9k). Nevertheless, only the serum metabolome and clinical data showed statistical significance.

#### 3.4.1 Integrative multi-omics network associations

**FIGURE 10:**
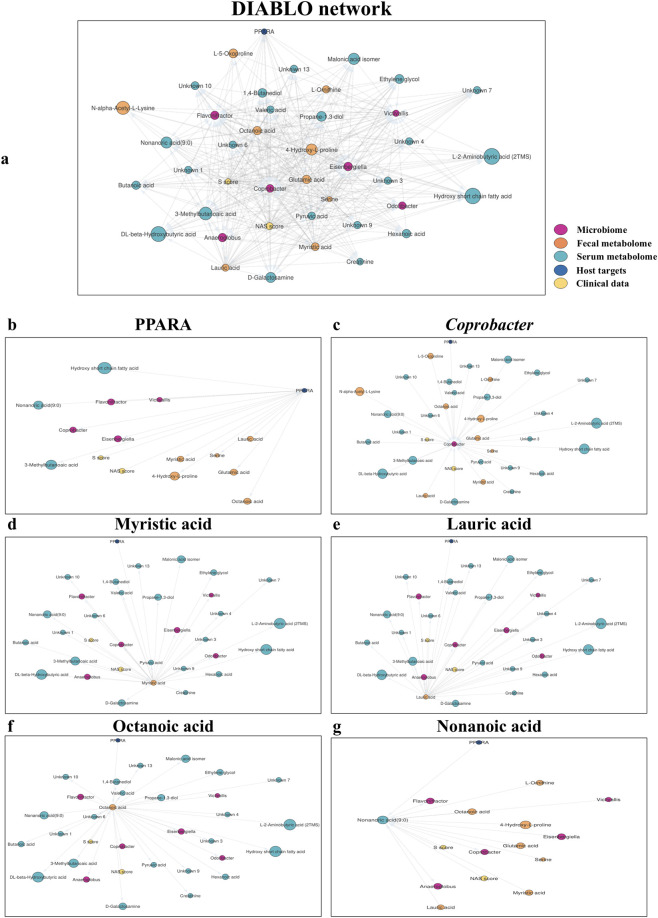
DIABLO generated networks. **(a)** Association network as depicted by DIABLO analysis for all variables suggested to be discriminatory along component 1 (*r* > 0.75). **(b–g)** Association subnetwork for **(b)** PPARA, **(c)** *Coprobacter*, **(d)** myristic acid, **(e)** lauric acid, **(f)** octanoic acid, and **(g)** nonanoic acid, as depicted by the DIABLO model. Microbiome nodes are colored in magenta, fecal metabolome nodes are colored in orange, serum metabolome nodes are colored in dark turquoise, host targets nodes are colored in dark blue, and clinical data nodes are colored in yellow. The arrowheads point toward the target.

The integrative multi-omics network depicted by our DIABLO model (Figure 10; Supplementary Table S59) included 34 nodes and 265 edges (a *.gml file for the network is shown in Supplementary Material, Figure 10a). When we retrieved the subnetworks for PPARA (at an *r* > 0.75), it exhibited no associations with serum metabolites (Figure 10b). PPARA was associated with fecal lauric, myristic, octanoic, and glutamic acids and 4-hydroxy-L-proline (Figure 10b).

Interestingly, fecal myristic, lauric, and octanoic acids showed identical interactions with the same serum metabolites, including nonanoic acid, the same bacterial genera, PPARA, steatosis grade (S score), and NAS scores, yet no interaction with other fecal metabolites (Figures 10d–f).

Serum nonanoic acid was predicted to interact with fecal metabolites, including myristic, lauric, octanoic, and glutamic acids and 4-hydroxy-L-proline (Figure 10g). In addition, nonanoic acid was predicted to interact with the bacterial genera showing associations with myristic, lauric, and octanoic acids: *Victivallis*, *Eisenbergiella*, *Flavonifractor*, and *Coprobacter*, which were also associated with PPARA (Figure 10g). All were associated with steatosis grade (S score) and NAS values (Figures 10b–g). It is worth mentioning that glutamic acid and 4-hydroxy-L-proline showed identical networks to myristic, lauric, and octanoic acids (Supplementary Figures S12a,b).

Lauric acid (LA)’s role in MASLD is controversial; one study reported that supplementing HFD with LA in mice increased liver triglycerides (Saraswathi et al., 2020), thereby suggesting detrimental effects of LA on the liver. However, another study showed that HFD supplemented with LA in MASLD rats improved liver histopathological picture (Sedik et al., 2024).

Octanoic acid alone or with LA could improve hepatic insulin sensitivity, yet octanoic acid could impair mitochondrial function, in a similar way to the lipotoxic palmitic acid (Rial et al., 2018). Octanoic acid is also a weak activator of PPARA (Intrasuksri et al., 1998). C3A cells exposed to excessive energy substrates (lactate, octanoate, and pyruvate) increased triglyceride accumulation and reactive oxygen species production. These effects were counteracted by the methyl-donor choline, reversing PPARA gene promoter methylation and thereby upregulating PPARA expression (Zhu et al., 2014). Additionally, treatment with lauric and myristic acids enhanced PPARA in HepG2 cells (Popeijus et al., 2014).

Our networks also predicted an impact by the TFs on the microbiome; this is echoed in a study in which PPARδ knockout in mice exacerbated HFD-induced MASLD and altered several bacterial genera (Wang et al., 2023).

To our knowledge, there are no reports about nonanoic acid in either MASLD or in any other disease. The mechanistic and causal contribution of nonanoic acid would need to be further elucidated.

Another interesting observation was about *Coprobacter* (Figure 10c)*,* which has the highest loading value in the microbiome component (Figure 9A). It was highly correlated (*r* > 0.9) with fecal lauric, myristic, octanoic, and glutamic acids and 4-hydroxy-L-proline, as well as steatosis grade (S score) and NAS values (Supplementary Figure S12C).

Ultimately, we inspected how different metabolites can affect the studied host proteins. Accordingly, we investigated, using SwissTargetPrediction, whether the 10 fecal and the top 15 serum metabolites suggested by the DIABLO model could potentially bind to SIRT1, FXR, PPARA, and PPARG (Supplementary Table S58). Myristic and lauric acids were predicted to bind PPARA with 85% and 40% probability, respectively. Interestingly, octanoic acid was predicted to bind both PPARA and FXR with 22% and 15% probability, respectively. Nonanoic acid was also anticipated to bind both PPARA and FXR with 16% and 11% probability, respectively.

## 4 Discussion

The subcellular localization of SIRT1, FXR, PPARA, and PPARG is important in disease pathogenesis (Byles et al., 2010; Andersen et al., 2011; Song et al., 2014; Shao et al., 2020; Cui et al., 2023; Zhong et al., 2023). While several *in vitro* and *in vivo* studies have shown that these proteins can be affected by the gut microbiome or its metabolites (Kelly et al., 2004; Avila-Roman et al., 2018; Chen et al., 2018; Wu et al., 2021; Nian et al., 2023; Zhong et al., 2023), these associations have not been previously investigated in humans. The gut microbiome is linked to the onset of MASLD, evidenced by clinical trials in which FMT improved MASLD (Le Roy et al., 2013; Hoyles et al., 2018; Aron-Wisnewsky et al., 2020; Xue L. et al., 2022). Additionally, diet-induced changes in the mouse microbiome partly contributed to persistent changes in the expression of key liver genes, even after reversal to the normal diet (Kim et al., 2019), highlighting a role for the gut microbiome and its metabolome in affecting the host proteins and thus disease pathogenesis. Nguyen et al. (2015) reported a discrepancy between human and mouse gut microbiomes, which limits the generalizability of mouse models. Therefore, we aimed to investigate possible associations between the subcellular localization of SIRT1, FXR, PPARA, and PPARG in the livers of patients with MASLD in relation to their gut microbiome composition and serum/stool metabolome through integrative analysis and to identify a potential multi-omics signature differentiating MASLD from MASH.

Our data reveal several associations between the gut microbiome and metabolome. Prominently, our microbiome analysis models suggested *Eubacterium* to discriminate not only between patients with MASLD and healthy controls, but also between patients with MASLD and MASH. *Eubacterium* was also positively associated with 15 fecal metabolites (including cholesterol, homogentisate, nonanoic acid, benzylalcohol, and N-acetylputrescine), and negatively associated with 18 fecal metabolites (including phenylalanine, 5-aminovaleric acid, putrescine, and inositol) and 13 serum metabolites. Most associated serum metabolites are amino acids, including glutamic and uric acids, phenylalanine, and tyrosine. Many of the *Eubacterium-*associated serum metabolites were enriched in patients compared to controls. *Eubacterium* prediction in our microbiome models and its high number of associations with metabolites propose an important role for this genus. There are controversial reports about *Eubacterium* in MASLD; however, several *Eubacterium* species are suggested to have an anti-inflammatory role (Schwenger et al., 2024). Additionally, FMT of patients with MASLD increased *Eubacterium siraeum*, which was negatively correlated with DNA methylation (Stols-Goncalves et al., 2023). Finally, *Eubacterium* was linked to several human diseases and to BA and cholesterol modifications in the gut (Gerard, 2013; Mukherjee et al., 2020). Multiple members of this genus are butyrate-producing with important roles in colonic motility, energy homeostasis, inflammation suppression, and gut immunomodulation (Mukherjee et al., 2020). These data highlight the role of the microbiome in disease pathogenesis and in regulating the host genes and protein expression.

Another unique bacterial genus, *Caloramator*, was depleted in patients with MASH and appeared in all correlations and multi-omics analyses. The exact role of this genus in MASLD requires further investigation because it has never been reported. A single study found *Caloramator* depleted in drug-induced liver injury (Rodriguez-Diaz et al., 2022). Another study reported two *Caloramator* species to degrade amino acids, such as glutamate, aspartate, arginine, methionine, histidine, threonine, and branched-chain amino acids, as well as carbohydrates such as glucose, fructose, cellobiose, mannose, and starch into acetate, lactate, ethanol, formate, and hydrogen (Tarlera et al., 1997; Plugge et al., 2000). This agrees with our results because most of these metabolites were altered in our patient cohort compared to controls.

To the best of our knowledge, we show for the first time that subcellular localization of the protein targets is correlated with gut microbial genera or metabolites and could potentially be predicted by them. SIRT1 nuclear localization was more associated with *Faecalibacterium*, *Phocaeicola*, and *Clostridium XlVa*, while its cytoplasmic localization was associated with *Paraprevotella*, *Desulfovibrio*, *Selenomonas*, *Collinsella*, and *Clostridium III*. Our results are supported by animal model findings, associating *Faecalibacterium*, *Clostridium, Desulfovibrio*, and SIRT1 expression (Li et al., 2018; Kim et al., 2022; Ren et al., 2022; Abd El-Emam et al., 2023; Yin et al., 2024); however, these studies investigated SIRT1 expression, not its subcellular localization. To our knowledge, no other bacterial genera associated with SIRT1 have been reported before. The nuclear localization of FXR, which is critical for its function in MASLD (Cui et al., 2023), was associated with fecal iditol, inositol, serum inositol-4-monophosphate, and chenodeoxycholic acid. On the other hand, cytoplasmic localization of FXR was associated with fecal homogentisate and GABA, as well as serum nonanoic and malonic acids and 2,2-dimethyl-1-pentanol. The association of these metabolites with FXR’s nuclear localization further reinforces the possible role of inositol as a protectant in MASLD (Pani et al., 2020) and emphasizes how BAs (like chenodeoxycholic acid) affect FXR (Cao et al., 2021). Unfortunately, the correlation models could not predict associations between the gut microbiome and metabolome with PPARA and PPARG.

The NAS, which differentiates between patients with MASH and MASLD, was positively correlated with *Rothia*, which was also positively correlated with the steatosis grade (S score) in our results. One study listed *Rothia* among 12 other genera that distinguish MASLD severity (Plaza-Diaz et al., 2020). The NAS was also positively correlated with *Acetatifactor*, which was negatively correlated with HbA1c%. Only one study reported the enrichment of *Acetatifactor* in HFD mice with no further characterization (Zhu et al., 2023). *Haemophilus*, similarly associated with NAS, was previously reported to be correlated with MASLD severity, but in the context of the oral microbiome (Zeybel et al., 2022). The gold standard for MASH diagnosis is a histopathological analysis of liver biopsy, which is invasive, expensive, and carries a risk of complications; hence, the attempts to find non-invasive alternatives that are specific and sensitive (Piazzolla and Mangia, 2020). Our logistic regression model, which differentiates between MASLD and MASH using the bacterial genera *Rothia*, *Acetatifactor*, and *Haemophilus*, which are highly correlated with NAS, yielded a promising model that will require further validation.

Another way to differentiate between patients with MASLD and MASH is integrated multi-omics analysis. Our DIABLO model suggested a signature of 20 microbial genera, 10 fecal and 30 serum metabolites, PPARA, PPARG, and SIRT1, and seven clinical attributes to be discriminatory. Among the suggested metabolites, myristic, lauric, octanoic, and nonanoic acids were computationally predicted to potentially bind PPARA and FXR. Interestingly, the same three fecal metabolites (myristic, lauric, and octanoic acids) had near-identical interaction networks with four bacterial genera (*Victivallis*, *Eisenbergiella*, *Flavonifractor*, and *Coprobacter*), serum nonanoic acid, and PPARA, as well as NAS and steatosis grade (S score). Among these bacterial genera is *Victivallis*, which was suggested by another integrative network analysis to be negatively correlated with hepatic steatosis (Zeybel et al., 2022). It is worth mentioning that those metabolites were also correlated with the nuclear or cytoplasmic expression of PPARA, PPARG, and FXR in our correlation analysis. Myristic and octanoic acids are involved in protein acylation, which can affect protein function, protein–protein interaction, and subcellular localization (Resh, 2016), which again must be explored in a MASLD context. Another important genus in our model is *Coprobacter*, which had the highest loading value in the microbiome component and strong correlations with fecal metabolites, NAS and steatosis grade (S score), and PPARA. *Coprobacter* was found to be dysregulated recently in a Chilean cohort of patients with MASLD (Zazueta et al., 2024), yet there were no mechanistic reports about its function, which must be further explored.

Our study shows strong correlations between lauric, myristic, octanoic, and nonanoic acids with the subcellular localization of PPARA and PPARG. In addition, the impact of the three fecal metabolites (myristic, lauric, and octanoic acids) on PPAR depicted computationally and by the DIABLO model was previously reported *in vitro*. For instance, lauric acid induced PPARG expression in macrophages, improving insulin sensitivity (Tham et al., 2020). Moreover, octanoic acid is also a weak activator of PPARA (Intrasuksri et al., 1998). C3A cells exposed to excessive energy substrates (lactate, octanoate, and pyruvate) increased triglyceride accumulation and reactive oxygen species production. These effects were counteracted by the methyl-donor choline reversing PPARA gene promoter methylation, thereby upregulating PPARA expression (Zhu et al., 2014). Pre-adipocyte treatment with octanoate or laurate induced PPARG expression, increasing lipid accumulation (Yang et al., 2009). Additionally, treatment with lauric and myristic acids enhanced PPARA in HepG2 cells (Popeijus et al., 2014). There are no reports about nonanoic acid in either MASLD or in any other disease, to our knowledge. Further mechanistic and causal experiments are needed to explore the impact of these fatty acids on the subcellular localization of PPARs.

Multi-omics and multivariate studies are challenging and complex because of numerous interdependent variables, which is a limitation, especially with a small sample size. Although we attempted to avoid over-fitting the models, we acknowledge that this is a pilot study that needs multi-center validation on larger cohorts to allow the generalizability of the identified signature. Another limitation of the study is the use of 16S amplicon sequencing, which restricts taxonomic identification to the genus level, without species or strain-level identification. This is especially important as some species can be commensal while others can be pathogenic. 16S rRNA microbiome profiling focuses on taxonomic assignment but is limited in predicting metabolic potential; this was compensated for and complemented by the use of metabolomics. It is also important to note, when interpreting our findings, that correlation analyses do not explain causal relationships. Further rigorous studies will be needed to establish causality; however, most such studies are usually in animal models, which are also not fully generalizable to human phenotypes. Mendelian randomization is another alternative but requires larger cohorts.

We suggest validating our results on a larger cohort of patients with the same ethnicity and dietary habits and examining how dietary habits can contribute to our findings. In addition, investigating microbial genera or metabolites correlated with other co-occurrences, such as diabetes or hypertension, would give a better insight into the disease. We also suggest exploring the causal or mechanistic involvement of metabolites or microbial genera on SIRT1, FXR, PPARA, and PPARG expression and subcellular localization and function in *in vitro* or *in vivo* models. It would be interesting to compare our findings to other cohorts with different ethnicities and dietary habits, which are two major factors complicating the understanding of this multifaceted disease.

Despite the abovementioned limitations, multiple significant correlations between the subcellular localization of important hepatoprotective liver proteins and microbial genera and metabolites were depicted in our cohort of patients with MASLD. We propose a discriminatory multi-omics signature between patients with MASLD and MASH for further validation.

## Data Availability

The 16S rRNA amplicon sequencing data are deposited in the Sequence Read Archive (SRA) NCBI repository under accession PRJNA1238093. Other datasets used and analyzed during the current study are provided in the Supplementary Tables. All codes used for analyses to generate the results are available at https://github.com/shereen-90/MASLD_multi-omics.git.

## Glossary

AUC: Area under the curve
BA: Bile acid
cytFXR: Cytoplasmic FXR
cytPPAR: Cytoplasmic PPAR
cytSIRT1: Cytoplasmic SIRT1
DIABLO: Data Integration Analysis for Biomarker discovery using Latent variable approaches for Omics studies
FA: Fatty acid
FFPE: Formalin-fixed paraffin-embedded
FMT: Fecal microbiota transplantation
FXR: Farnesoid X receptor
GABA: Gamma-aminobutyric acid
GC/MS: Gas chromatography/mass spectrometry
Hb1Ac%: Hemoglobin A1C
HDL: High-density lipoprotein
HFD: High-fat diet
IHC: Immunohistochemistry
LA: Lauric acid
LDL: Low-density lipoprotein
MASH: Metabolic-associated steatohepatitis
MASLD: Metabolic dysfunction-associated steatotic liver disease
NAS: NAFLD activity score
OPLS-DA: Orthogonal partial least square-discriminant analysis
P.T%: Prothrombin time
PCA: Principal component analysis
PPARs: Peroxisome proliferation-associated receptors
ROC: Receiver-operator characteristic
SAF score: Steatosis, activity, fibrosis score
SIRT1: Sirtuin1
TF: Transcription factor
TMAVA: N,N,N-trimethyl-5-aminovaleric acid
TyG index: Triglyceride-glucose index

## References

[B1] Abd El-Emam M. M. Mostafa M. Farag A. A. Youssef H. S. El-Demerdash A. S. Bayoumi H. (2023). The potential effects of quercetin-loaded nanoliposomes on amoxicillin/clavulanate-induced hepatic damage: targeting the SIRT1/Nrf2/NF-kappaB signaling pathway and microbiota modulation. Antioxidants (Basel) 12, 8. 10.3390/antiox12081487 PMC10451903 37627483

[B2] Andersen J. L. Thompson J. W. Lindblom K. R. Johnson E. S. Yang C. S. Lilley L. R. (2011). A biotin switch-based proteomics approach identifies 14-3-3zeta as a target of Sirt1 in the metabolic regulation of caspase-2. Mol. Cell. 43 (5), 834–842. 10.1016/j.molcel.2011.07.028 21884983 PMC3417139

[B3] Aron-Wisnewsky J. Vigliotti C. Witjes J. Le P. Holleboom A. G. Verheij J. (2020). Gut microbiota and human NAFLD: disentangling microbial signatures from metabolic disorders. Nat. Rev. Gastroenterol. Hepatol. 17 (5), 279–297. 10.1038/s41575-020-0269-9 32152478

[B4] Avila-Roman J. Talero E. de Los Reyes C. Garcia-Maurino S. Motilva V. (2018). Microalgae-derived oxylipins decrease inflammatory mediators by regulating the subcellular location of NFkappaB and PPAR-gamma. Pharmacol. Res. 128, 220–230. 10.1016/j.phrs.2017.10.009 29129670

[B5] Berthier A. Johanns M. Zummo F. P. Lefebvre P. Staels B. (2021). PPARs in liver physiology. Biochim. Biophys. Acta Mol. Basis Dis. 1867 (5), 166097. 10.1016/j.bbadis.2021.166097 33524529

[B6] Boccuto L. Tack J. Ianiro G. Abenavoli L. Scarpellini E. (2023). Human genes involved in the interaction between host and gut microbiome: regulation and pathogenic mechanisms. Genes (Basel) 14 (4), 857. 10.3390/genes14040857 37107615 PMC10137629

[B7] Boursier J. Mueller O. Barret M. Machado M. Fizanne L. Araujo-Perez F. (2016). The severity of nonalcoholic fatty liver disease is associated with gut dysbiosis and shift in the metabolic function of the gut microbiota. Hepatology 63 (3), 764–775. 10.1002/hep.28356 26600078 PMC4975935

[B8] Byles V. Chmilewski L. K. Wang J. Zhu L. Forman L. W. Faller D. V. (2010). Aberrant cytoplasm localization and protein stability of SIRT1 is regulated by PI3K/IGF-1R signaling in human cancer cells. Int. J. Biol. Sci. 6 (6), 599–612. 10.7150/ijbs.6.599 20941378 PMC2952410

[B9] Cao S. Meng X. Li Y. Sun L. Jiang L. Xuan H. (2021). Bile acids elevated in chronic periaortitis could activate farnesoid-X-receptor to suppress IL-6 production by macrophages. Front. Immunol. 12, 632864. 10.3389/fimmu.2021.632864 33968024 PMC8100322

[B10] Chen Y. T. Lin Y. C. Lin J. S. Yang N. S. Chen M. J. (2018). Sugary kefir strain *Lactobacillus Mali* APS1 ameliorated hepatic steatosis by regulation of SIRT-1/Nrf-2 and gut microbiota in rats. Mol. Nutr. Food Res. 62 (8), e1700903. 10.1002/mnfr.201700903 29508520

[B11] Clifford B. L. Sedgeman L. R. Williams K. J. Morand P. Cheng A. Jarrett K. E. (2021). FXR activation protects against NAFLD *via* bile-acid-dependent reductions in lipid absorption. Cell Metab. 33 (8), 1671–1684 e1674. 10.1016/j.cmet.2021.06.012 34270928 PMC8353952

[B12] Cui S. Hu H. Chen A. Cui M. Pan X. Zhang P. (2023). SIRT1 activation synergizes with FXR agonism in hepatoprotection *via* governing nucleocytoplasmic shuttling and degradation of FXR. Acta Pharm. Sin. B 13 (2), 559–576. 10.1016/j.apsb.2022.08.019 36873184 PMC9978964

[B13] Dahl W. J. Rivero Mendoza D. Lambert J. M. (2020). Diet, nutrients and the microbiome. Prog. Mol. Biol. Transl. Sci. 171, 237–263. 10.1016/bs.pmbts.2020.04.006 32475524

[B14] Erben V. Poschet G. Schrotz-King P. Brenner H. (2021). Evaluation of different stool extraction methods for metabolomics measurements in human faecal samples. BMJ Nutr. Prev. Health 4 (2), 374–384. 10.1136/bmjnph-2020-000202 PMC8718864 35028509

[B15] Frau A. Lett L. Slater R. Young G. R. Stewart C. J. Berrington J. (2021). The stool volatile metabolome of pre-term babies. Molecules 26 (11), 3341. 10.3390/molecules26113341 34199338 PMC8199543

[B16] Gerard P. (2013). Metabolism of cholesterol and bile acids by the gut microbiota. Pathogens 3 (1), 14–24. 10.3390/pathogens3010014 25437605 PMC4235735

[B17] Gottlieb A. Leven A. S. Sowa J. P. Borucki K. Link A. Yilmaz E. (2021). Lipoprotein and metabolic profiles indicate similar cardiovascular risk of liver steatosis and NASH. Digestion 102 (5), 671–681. 10.1159/000510600 33080603

[B18] Hjorth M. F. Christensen L. Kjolbaek L. Larsen L. H. Roager H. M. Kiilerich P. (2020). Pretreatment *Prevotella-to-Bacteroides* ratio and markers of glucose metabolism as prognostic markers for dietary weight loss maintenance. Eur. J. Clin. Nutr. 74 (2), 338–347. 10.1038/s41430-019-0466-1 31285554

[B19] Hou X. Xu S. Maitland-Toolan K. A. Sato K. Jiang B. Ido Y. (2008). SIRT1 regulates hepatocyte lipid metabolism through activating AMP-activated protein kinase. J. Biol. Chem. 283 (29), 20015–20026. 10.1074/jbc.M802187200 18482975 PMC2459285

[B20] Hoyles L. Fernandez-Real J. M. Federici M. Serino M. Abbott J. Charpentier J. (2018). Molecular phenomics and metagenomics of hepatic steatosis in non-diabetic obese women. Nat. Med. 24 (7), 1070–1080. 10.1038/s41591-018-0061-3 29942096 PMC6140997

[B21] Hu Y. Sun C. Chen Y. Liu Y. D. Fan J. G. (2024). Pipeline of new drug treatment for non-alcoholic fatty liver disease/metabolic dysfunction-associated steatotic liver disease. J. Clin. Transl. Hepatol. 12 (9), 802–814. 10.14218/JCTH.2024.00123 39280073 PMC11393841

[B22] Intrasuksri U. Rangwala S. M. O'Brien M. Noonan D. J. Feller D. R. (1998). Mechanisms of peroxisome proliferation by perfluorooctanoic acid and endogenous fatty acids. Gen. Pharmacol. 31 (2), 187–197. 10.1016/s0306-3623(98)00029-9 9688458

[B23] Keam S. J. (2024). Resmetirom: first approval. Drugs 84 (6), 729–735. 10.1007/s40265-024-02045-0 38771485

[B24] Kelly D. Campbell J. I. King T. P. Grant G. Jansson E. A. Coutts A. G. (2004). Commensal anaerobic gut bacteria attenuate inflammation by regulating nuclear-cytoplasmic shuttling of PPAR-gamma and RelA. Nat. Immunol. 5 (1), 104–112. 10.1038/ni1018 14691478

[B25] Kim H. Worsley O. Yang E. Purbojati R. W. Liang A. L. Tan W. (2019). Persistent changes in liver methylation and microbiome composition following reversal of diet-induced non-alcoholic-fatty liver disease. Cell Mol. Life Sci. 76 (21), 4341–4354. 10.1007/s00018-019-03114-4 31119300 PMC11105172

[B26] Kim D. Yan J. Bak J. Park J. Lee H. Kim H. (2022). *Sargassum thunbergii* extract attenuates high-fat diet-induced obesity in mice by modulating AMPK activation and the gut microbiota. Foods 11 (16), 2529. 10.3390/foods11162529 36010531 PMC9407432

[B27] Kleiner D. E. Brunt E. M. Van Natta M. Behling C. Contos M. J. Cummings O. W. (2005). Design and validation of a histological scoring system for nonalcoholic fatty liver disease. Hepatology 41 (6), 1313–1321. 10.1002/hep.20701 15915461

[B28] Le Roy T. Llopis M. Lepage P. Bruneau A. Rabot S. Bevilacqua C. (2013). Intestinal microbiota determines development of non-alcoholic fatty liver disease in mice. Gut 62 (12), 1787–1794. 10.1136/gutjnl-2012-303816 23197411

[B29] Lee H. An J. Kim J. Choi D. Song Y. Lee C. K. (2022). A novel bacterium, *Butyricimonas virosa*, preventing HFD-induced diabetes and metabolic disorders in mice *via* GLP-1 receptor. Front. Microbiol. 13, 858192. 10.3389/fmicb.2022.858192 35655996 PMC9152154

[B30] Li C. C. Liu C. Fu M. Hu K. Q. Aizawa K. Takahashi S. (2018). Tomato powder inhibits hepatic steatosis and inflammation potentially through restoring SIRT1 activity and adiponectin function independent of carotenoid cleavage enzymes in mice. Mol. Nutr. Food Res. 62 (8), e1700738. 10.1002/mnfr.201700738 29266812

[B31] Li F. Ye J. Shao C. Zhong B. (2021). Compositional alterations of gut microbiota in nonalcoholic fatty liver disease patients: a systematic review and meta-analysis. Lipids Health Dis. 20 (1), 22. 10.1186/s12944-021-01440-w 33637088 PMC7908766

[B32] Lin Z. Zhang R. Ren S. He T. Mi H. Jiang W. (2025). Global burden of metabolic dysfunction-associated steatotic liver disease from 1990 to 2021 and the prediction for the next 10 years. Prev. Med. Rep. 59, 103248. 10.1016/j.pmedr.2025.103248 41048597 PMC12495469

[B33] Liu L. Yin M. Gao J. Yu C. Lin J. Wu A. (2023). Intestinal barrier function in the pathogenesis of nonalcoholic fatty liver disease. J. Clin. Transl. Hepatol. 11 (2), 452–458. 10.14218/JCTH.2022.00089 36643028 PMC9817057

[B34] Lv X. Lv Y. Dai X. (2023). Lactate, histone lactylation and cancer hallmarks. Expert Rev. Mol. Med. 25, e7. 10.1017/erm.2022.42 36621008

[B35] Ma Y. L. Ke J. F. Wang J. W. Wang Y. J. Xu M. R. Li L. X. (2023). Blood lactate levels are associated with an increased risk of metabolic dysfunction-associated fatty liver disease in type 2 diabetes: a real-world study. Front. Endocrinol. (Lausanne) 14, 1133991. 10.3389/fendo.2023.1133991 37223022 PMC10200915

[B36] Mantovani A. Scorletti E. Mosca A. Alisi A. Byrne C. D. Targher G. (2020). Complications, morbidity and mortality of nonalcoholic fatty liver disease. Metabolism 111S, 154170. 10.1016/j.metabol.2020.154170 32006558

[B37] Mukherjee A. Lordan C. Ross R. P. Cotter P. D. (2020). Gut microbes from the phylogenetically diverse genus *Eubacterium* and their various contributions to gut health. Gut Microbes 12 (1), 1802866. 10.1080/19490976.2020.1802866 32835590 PMC7524325

[B38] Nguyen T. L. Vieira-Silva S. Liston A. Raes J. (2015). How informative is the mouse for human gut microbiota research? Dis. Model Mech. 8 (1), 1–16. 10.1242/dmm.017400 25561744 PMC4283646

[B39] Nian F. Wu L. Xia Q. Tian P. Ding C. Lu X. (2023). *Akkermansia muciniphila* and *Bifidobacterium bifidum* prevent NAFLD by regulating FXR expression and gut microbiota. J. Clin. Transl. Hepatol. 11 (4), 763–776. 10.14218/JCTH.2022.00415 37408808 PMC10318293

[B40] Nunez-Sanchez M. A. Martinez-Sanchez M. A. Sierra-Cruz M. Lambertos A. Rico-Chazarra S. Oliva-Bolarin A. (2024). Increased hepatic putrescine levels as a new potential factor related to the progression of metabolic dysfunction-associated steatotic liver disease. J. Pathol. 264 (1), 101–111. 10.1002/path.6330 39022853 PMC11300153

[B41] Oh K. K. Gupta H. Min B. H. Ganesan R. Sharma S. P. Won S. M. (2023). The identification of metabolites from gut microbiota in NAFLD *via* network pharmacology. Sci. Rep. 13 (1), 724. 10.1038/s41598-023-27885-w 36639568 PMC9839744

[B42] Pani A. Giossi R. Menichelli D. Fittipaldo V. A. Agnelli F. Inglese E. (2020). Inositol and non-alcoholic fatty liver disease: a systematic review on deficiencies and supplementation. Nutrients 12, 11. 10.3390/nu12113379 33153126 PMC7694137

[B43] Parker B. J. Wearsch P. A. Veloo A. C. M. Rodriguez-Palacios A. (2020). The genus *alistipes*: gut bacteria with emerging implications to inflammation, cancer, and mental health. Front. Immunol. 11, 906. 10.3389/fimmu.2020.00906 32582143 PMC7296073

[B44] Patel H. Truant R. Rachubinski R. A. Capone J. P. (2005). Activity and subcellular compartmentalization of peroxisome proliferator-activated receptor alpha are altered by the centrosome-associated protein CAP350. J. Cell Sci. 118 (Pt 1), 175–186. 10.1242/jcs.01600 15615782

[B45] Patel J. Sohal A. Bains K. Chaudhry H. Kohli I. Khanna T. (2024). Association of metabolic dysfunction-associated fatty liver disease with gastrointestinal infections: insights from National Inpatient Sample Database. BMJ Open Gastroenterol. 11 (1), e001224. 10.1136/bmjgast-2023-001224 PMC10870785 38237944

[B46] Pawlak M. Lefebvre P. Staels B. (2015). Molecular mechanism of PPARalpha action and its impact on lipid metabolism, inflammation and fibrosis in non-alcoholic fatty liver disease. J. Hepatol. 62 (3), 720–733. 10.1016/j.jhep.2014.10.039 25450203

[B47] Peng L. Yang H. Ye Y. Ma Z. Kuhn C. Rahmeh M. (2021). Role of peroxisome proliferator-activated receptors (PPARs) in trophoblast functions. Int. J. Mol. Sci. 22 (1), 433. 10.3390/ijms22010433 33406768 PMC7795665

[B48] Piazzolla V. A. Mangia A. (2020). Noninvasive diagnosis of NAFLD and NASH. Cells 9, 4. 10.3390/cells9041005 32316690 PMC7226476

[B49] Pineda Torra I. Claudel T. Duval C. Kosykh V. Fruchart J. C. Staels B. (2003). Bile acids induce the expression of the human peroxisome proliferator-activated receptor alpha gene *via* activation of the farnesoid X receptor. Mol. Endocrinol. 17 (2), 259–272. 10.1210/me.2002-0120 12554753

[B50] Plaza-Diaz J. Solis-Urra P. Rodriguez-Rodriguez F. Olivares-Arancibia J. Navarro-Oliveros M. Abadia-Molina F. (2020). The gut barrier, intestinal microbiota, and liver disease: molecular mechanisms and strategies to manage. Int. J. Mol. Sci. 21, 21. 10.3390/ijms21218351 33171747 PMC7664383

[B51] Plugge C. M. Zoetendal E. G. Stams A. J. (2000). *Caloramator coolhaasii* sp. nov., a glutamate-degrading, moderately thermophilic anaerobe. Int. J. Syst. Evol. Microbiol. 50 (Pt 3), 1155–1162. 10.1099/00207713-50-3-1155 10843058

[B52] Ponugoti B. Kim D. H. Xiao Z. Smith Z. Miao J. Zang M. (2010). SIRT1 deacetylates and inhibits SREBP-1C activity in regulation of hepatic lipid metabolism. J. Biol. Chem. 285 (44), 33959–33970. 10.1074/jbc.M110.122978 20817729 PMC2962496

[B53] Popeijus H. E. van Otterdijk S. D. van der Krieken S. E. Konings M. Serbonij K. Plat J. (2014). Fatty acid chain length and saturation influences PPARα transcriptional activation and repression in HepG2 cells. Mol. Nutr. Food Res. 58 (12), 2342–2349. 10.1002/mnfr.201400314 25255786

[B54] Purushotham A. Schug T. T. Xu Q. Surapureddi S. Guo X. Li X. (2009). Hepatocyte-specific deletion of SIRT1 alters fatty acid metabolism and results in hepatic steatosis and inflammation. Cell Metab. 9 (4), 327–338. 10.1016/j.cmet.2009.02.006 19356714 PMC2668535

[B55] Ren M. Li M. Y. Lu L. Q. Liu Y. S. An F. K. Huang K. (2022). *Arenga pinnata* resistant starch modulate gut microbiota and ameliorate intestinal inflammation in aged mice. Nutrients 14 (19), 3931. 10.3390/nu14193931 36235583 PMC9572357

[B56] Resh M. D. (2016). Fatty acylation of proteins: the long and the short of it. Prog. Lipid. Res. 63, 120–131. 10.1016/j.plipres.2016.05.002 27233110 PMC4975971

[B57] Rial S. A. Ravaut G. Malaret T. B. Bergeron K. F. Mounier C. (2018). Hexanoic, octanoic and decanoic acids promote basal and insulin-induced phosphorylation of the Akt-mTOR axis and a balanced lipid metabolism in the HepG2 hepatoma cell line. Molecules 23, 9. 10.3390/molecules23092315 30208604 PMC6225498

[B58] Rinella M. E. Lazarus J. V. Ratziu V. Francque S. M. Sanyal A. J. Kanwal F. (2024). A multisociety Delphi consensus statement on new fatty liver disease nomenclature. Ann. Hepatol. 29 (1), 101133. 10.1016/j.aohep.2023.101133 37364816

[B59] Rochon J. Kalinowski P. Szymanek-Majchrzak K. Grat M. (2024). Role of gut-liver axis and glucagon-like peptide-1 receptor agonists in the treatment of metabolic dysfunction-associated fatty liver disease. World J. Gastroenterol. 30 (23), 2964–2980. 10.3748/wjg.v30.i23.2964 38946874 PMC11212696

[B60] Rodriguez-Diaz C. Taminiau B. Garcia-Garcia A. Cueto A. Robles-Diaz M. Ortega-Alonso A. (2022). Microbiota diversity in nonalcoholic fatty liver disease and in drug-induced liver injury. Pharmacol. Res. 182, 106348. 10.1016/j.phrs.2022.106348 35817360

[B61] Saraswathi V. Kumar N. Gopal T. Bhatt S. Ai W. Ma C. (2020). Lauric acid *versus* palmitic acid: effects on adipose tissue inflammation, insulin resistance, and non-alcoholic fatty liver disease in obesity. Biol. (Basel) 9, 11. 10.3390/biology9110346 33105887 PMC7690582

[B62] Schwartz L. Supuran C. T. Alfarouk K. O. (2017). The Warburg effect and the hallmarks of cancer. Anticancer Agents Med. Chem. 17 (2), 164–170. 10.2174/1871520616666161031143301 27804847

[B63] Schwenger K. J. P. Sharma D. Ghorbani Y. Xu W. Lou W. Comelli E. M. (2024). Links between gut microbiome, metabolome, clinical variables and non-alcoholic fatty liver disease severity in bariatric patients. Liver Int. 44 (5), 1176–1188. 10.1111/liv.15864 38353022

[B64] Sedik A. A. Elgohary R. Khalifa E. Khalil W. K. B. H I. S. M B. S. (2024). Lauric acid attenuates hepato-metabolic complications and molecular alterations in high-fat diet-induced nonalcoholic fatty liver disease in rats. Toxicol. Mech. Methods 34 (4), 454–467. 10.1080/15376516.2023.2301344 38166588

[B65] Shao W. Kuhn C. Mayr D. Ditsch N. Kailuwait M. Wolf V. (2020). Cytoplasmic PPARgamma is a marker of poor prognosis in patients with Cox-1 negative primary breast cancers. J. Transl. Med. 18 (1), 94. 10.1186/s12967-020-02271-6 32085795 PMC7035771

[B66] Song S. Luo M. Song Y. Liu T. Zhang H. Xie Z. (2014). Prognostic role of SIRT1 in hepatocellular carcinoma. J. Coll. Physicians Surg. Pak. 24 (11), 849–854. 25404446

[B67] Stols-Goncalves D. Mak A. L. Madsen M. S. van der Vossen E. W. J. Bruinstroop E. Henneman P. (2023). Faecal microbiota transplantation affects liver DNA methylation in non-alcoholic fatty liver disease: a multi-omics approach. Gut Microbes 15 (1), 2223330. 10.1080/19490976.2023.2223330 37317027 PMC10269428

[B68] Sun P. Q. Dong W. M. Yuan Y. F. Cao Q. Chen X. Y. Guo L. L. (2024). Targeted metabolomics study of fatty-acid metabolism in lean metabolic-associated fatty liver disease patients. World J. Gastroenterol. 30 (27), 3290–3303. 10.3748/wjg.v30.i27.3290 39086751 PMC11287418

[B69] Tacke F. Horn P. Wong V. W. S. Ratziu V. Bugianesi E. Francque S. (2024). EASL-EASD-EASO Clinical Practice Guidelines on the management of metabolic dysfunction-associated steatotic liver disease (MASLD). J. Hepatol. 81 (3), 492–542. 10.1016/j.jhep.2024.04.031 38851997

[B70] Tarlera S. Muxi L. Soubes M. Stams A. J. (1997). *Caloramator proteoclasticus* sp. Nov., a new moderately thermophilic anaerobic proteolytic bacterium. Int. J. Syst. Bacteriol. 47 (3), 651–656. 10.1099/00207713-47-3-651 9226895

[B71] Tham Y. Y. Choo Q. C. Muhammad T. S. T. Chew C. H. (2020). Lauric acid alleviates insulin resistance by improving mitochondrial biogenesis in THP-1 macrophages. Mol. Biol. Rep. 47 (12), 9595–9607. 10.1007/s11033-020-06019-9 33259010

[B72] Thomsen K. L. Eriksen P. L. Kerbert A. J. De Chiara F. Jalan R. Vilstrup H. (2023). Role of ammonia in NAFLD: an unusual suspect. JHEP Rep. 5 (7), 100780. 10.1016/j.jhepr.2023.100780 37425212 PMC10326708

[B73] Tian C. Huang R. Xiang M. (2024). SIRT1: harnessing multiple pathways to hinder NAFLD. Pharmacol. Res. 203, 107155. 10.1016/j.phrs.2024.107155 38527697

[B74] Vallianou N. Christodoulatos G. S. Karampela I. Tsilingiris D. Magkos F. Stratigou T. (2021). Understanding the role of the gut microbiome and microbial metabolites in non-alcoholic fatty liver disease: current evidence and perspectives. Biomolecules 12 (1), 56. 10.3390/biom12010056 35053205 PMC8774162

[B75] Wang Y. T. Wang F. F. Li H. Xu J. Y. Lu X. L. Wang Y. (2023). Deletion of the PPARdelta gene exacerbates high-fat diet-induced nonalcoholic fatty liver disease in mice through the gut-liver axis. Cell. Mol. Biol. (Noisy-le-grand) 69 (10), 121–128. 10.14715/cmb/2023.69.10.17 37953575

[B76] Wu G. D. Chen J. Hoffmann C. Bittinger K. Chen Y. Y. Keilbaugh S. A. (2011). Linking long-term dietary patterns with gut microbial enterotypes. Science 334 (6052), 105–108. 10.1126/science.1208344 21885731 PMC3368382

[B77] Wu L. Li J. Feng J. Ji J. Yu Q. Li Y. (2021). Crosstalk between PPARs and gut microbiota in NAFLD. Biomed. Pharmacother. 136, 111255. 10.1016/j.biopha.2021.111255 33485064

[B78] Xue L. Deng Z. Luo W. He X. Chen Y. (2022a). Effect of fecal microbiota transplantation on non-alcoholic fatty liver disease: a randomized clinical trial. Front. Cell. Infect. Microbiol. 12, 759306. 10.3389/fcimb.2022.759306 35860380 PMC9289257

[B79] Xue Y. Xu J. Li M. Gao Y. (2022b). Potential screening indicators for early diagnosis of NAFLD/MAFLD and liver fibrosis: triglyceride glucose index-related parameters. Front. Endocrinol. (Lausanne) 13, 951689. 10.3389/fendo.2022.951689 36120429 PMC9478620

[B80] Yang K. Song M. (2023). New insights into the pathogenesis of metabolic-associated fatty liver disease (MAFLD): gut-liver-heart crosstalk. Nutrients 15 (18), 3970. 10.3390/nu15183970 37764755 PMC10534946

[B81] Yang M. Zhang C. Y. (2021). G protein-coupled receptors as potential targets for nonalcoholic fatty liver disease treatment. World J. Gastroenterol. 27 (8), 677–691. 10.3748/wjg.v27.i8.677 33716447 PMC7934005

[B82] Yang J. Y. Della-Fera M. A. Rayalam S. Park H. J. Ambati S. Hausman D. B. (2009). Regulation of adipogenesis by medium-chain fatty acids in the absence of hormonal cocktail. J. Nutr. Biochem. 20 (7), 537–543. 10.1016/j.jnutbio.2008.05.013 18789670

[B83] Yang Y. Misra B. B. Liang L. Bi D. Weng W. Wu W. (2019). Integrated microbiome and metabolome analysis reveals a novel interplay between commensal bacteria and metabolites in colorectal cancer. Theranostics 9 (14), 4101–4114. 10.7150/thno.35186 31281534 PMC6592169

[B84] Yang L. Dai Y. He H. Liu Z. Liao S. Zhang Y. (2022a). Integrative analysis of gut microbiota and fecal metabolites in metabolic associated fatty liver disease patients. Front. Microbiol. 13, 969757. 10.3389/fmicb.2022.969757 36071958 PMC9441872

[B85] Yang Y. Y. Xie L. Zhang N. P. Zhou D. Liu T. T. Wu J. (2022b). Updates on novel pharmacotherapeutics for the treatment of nonalcoholic steatohepatitis. Acta Pharmacol. Sin. 43 (5), 1180–1190. 10.1038/s41401-022-00860-3 35190696 PMC9061746

[B86] Yin Y. Gong S. Han M. Wang J. Shi H. Jiang X. (2024). Leucine regulates lipid metabolism in adipose tissue through adipokine-mTOR-SIRT1 signaling pathway and bile acid-microbe axis in a finishing pig model. Anim. Nutr. 16, 158–173. 10.1016/j.aninu.2023.10.005 38357569 PMC10864217

[B87] Zazueta A. Valenzuela-Perez L. Ortiz-Lopez N. Pinto-Leon A. Torres V. Guinez D. (2024). Alteration of gut microbiota composition in the progression of liver damage in patients with metabolic dysfunction-associated steatotic liver disease (MASLD). Int. J. Mol. Sci. 25 (8), 4387. 10.3390/ijms25084387 38673972 PMC11050088

[B88] Zeybel M. Arif M. Li X. Altay O. Yang H. Shi M. (2022). Multiomics analysis reveals the impact of microbiota on host metabolism in hepatic steatosis. Adv. Sci. (Weinh) 9 (11), e2104373. 10.1002/advs.202104373 35128832 PMC9008426

[B89] Zhao M. Zhao L. Xiong X. He Y. Huang W. Liu Z. (2020). TMAVA, a metabolite of intestinal microbes, is increased in plasma from patients with liver steatosis, inhibits gamma-butyrobetaine hydroxylase, and exacerbates fatty liver in mice. Gastroenterology 158 (8), 2266–2281 e2227. 10.1053/j.gastro.2020.02.033 32105727

[B90] Zhong J. He X. Gao X. Liu Q. Zhao Y. Hong Y. (2023). Hyodeoxycholic acid ameliorates nonalcoholic fatty liver disease by inhibiting RAN-mediated PPARalpha nucleus-cytoplasm shuttling. Nat. Commun. 14 (1), 5451. 10.1038/s41467-023-41061-8 37673856 PMC10482907

[B91] Zhou D. Pan Q. Shen F. Cao H. X. Ding W. J. Chen Y. W. (2017). Total fecal microbiota transplantation alleviates high-fat diet-induced steatohepatitis in mice *via* beneficial regulation of gut microbiota. Sci. Rep. 7 (1), 1529. 10.1038/s41598-017-01751-y 28484247 PMC5431549

[B92] Zhu J. Wu Y. Tang Q. Leng Y. Cai W. (2014). The effects of choline on hepatic lipid metabolism, mitochondrial function and antioxidative status in human hepatic C3A cells exposed to excessive energy substrates. Nutrients 6 (7), 2552–2571. 10.3390/nu6072552 25010553 PMC4113756

[B93] Zhu X. Cai J. Wang Y. Liu X. Chen X. Wang H. (2023). A high-fat diet increases the characteristics of gut microbial composition and the intestinal damage associated with non-alcoholic fatty liver disease. Int. J. Mol. Sci. 24 (23), 16733. 10.3390/ijms242316733 38069055 PMC10706137

