# Supplementary material for: Association between the subcellular localization of host proteins and gut microbiome and metabolome in metabolic dysfunction-associated steatotic liver disease: a pilot study
Source: Front Mol Biosci. 2026 Jul 2;13:1703547. doi: 10.3389/fmolb.2026.1703547 (PMC13372696; doi:10.3389/fmolb.2026.1703547)
Supplement: Supplementary file 1 [file DataSheet1.pdf]

Supplementary Figures

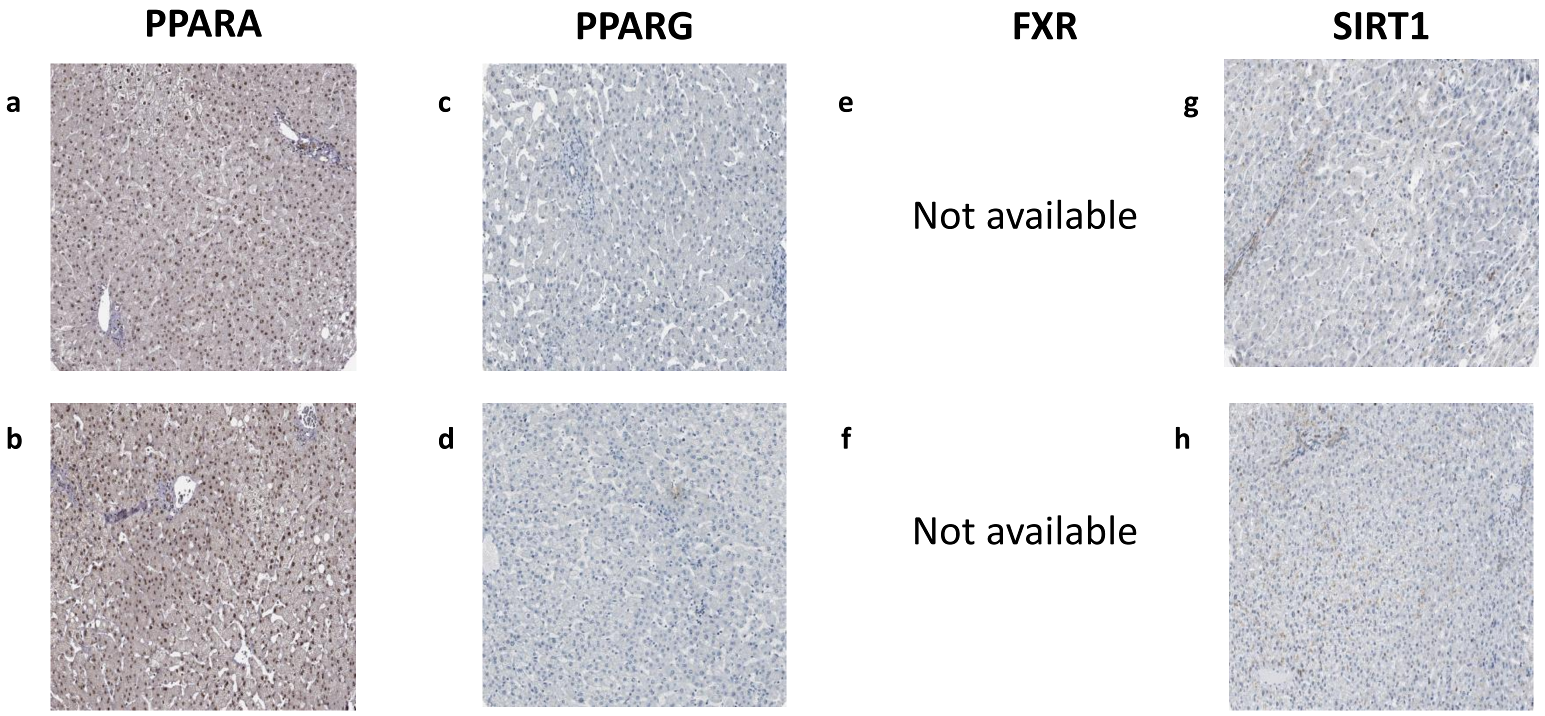

Supplementary Figure S1. Representative IHC images retrieved from the Human Protein Atlas database (https://www.proteinatlas.org/) for normal liver tissues stained for PPARA, PPARRG and SIRT1. a) PPARA staining from a male (patient ID: 1720); with moderate hepatocyte staining (>75%) and nuclear localization (https://www.proteinatlas.org/ENSG00000186951-PPARA/tissue/liver#img). b) PPARA staining from a female (patient ID: 3402); with moderate hepatocyte staining (>75%) and nuclear localization (https://www.proteinatlas.org/ENSG00000186951-PPARA/tissue/liver#img). c) PPARG staining from a male (patient ID: 1720); with negative hepatocyte staining (not detected) (https://www.proteinatlas.org/ENSG00000132170-PPARG/tissue/liver#img). d) PPARG staining from a female (patient ID: 1899); with negative hepatocyte staining (not detected) (https://www.proteinatlas.org/ENSG00000132170-PPARG/tissue/liver#img). (e&f) No available tissues expression for FXR (NR1H4). g) SIRT1 staining from a male (patient ID: 1720); with low hepatocyte staining (<25%) and cytoplasmic/membranous localization (https://www.proteinatlas.org/ENSG00000096717-SIRT1/tissue/liver#img). h) SIRT1 staining from a female (patient ID: 1899); with low hepatocyte staining (<25%) and cytoplasmic/membranous localization (https://www.proteinatlas.org/ENSG00000096717-SIRT1/tissue/liver#img).

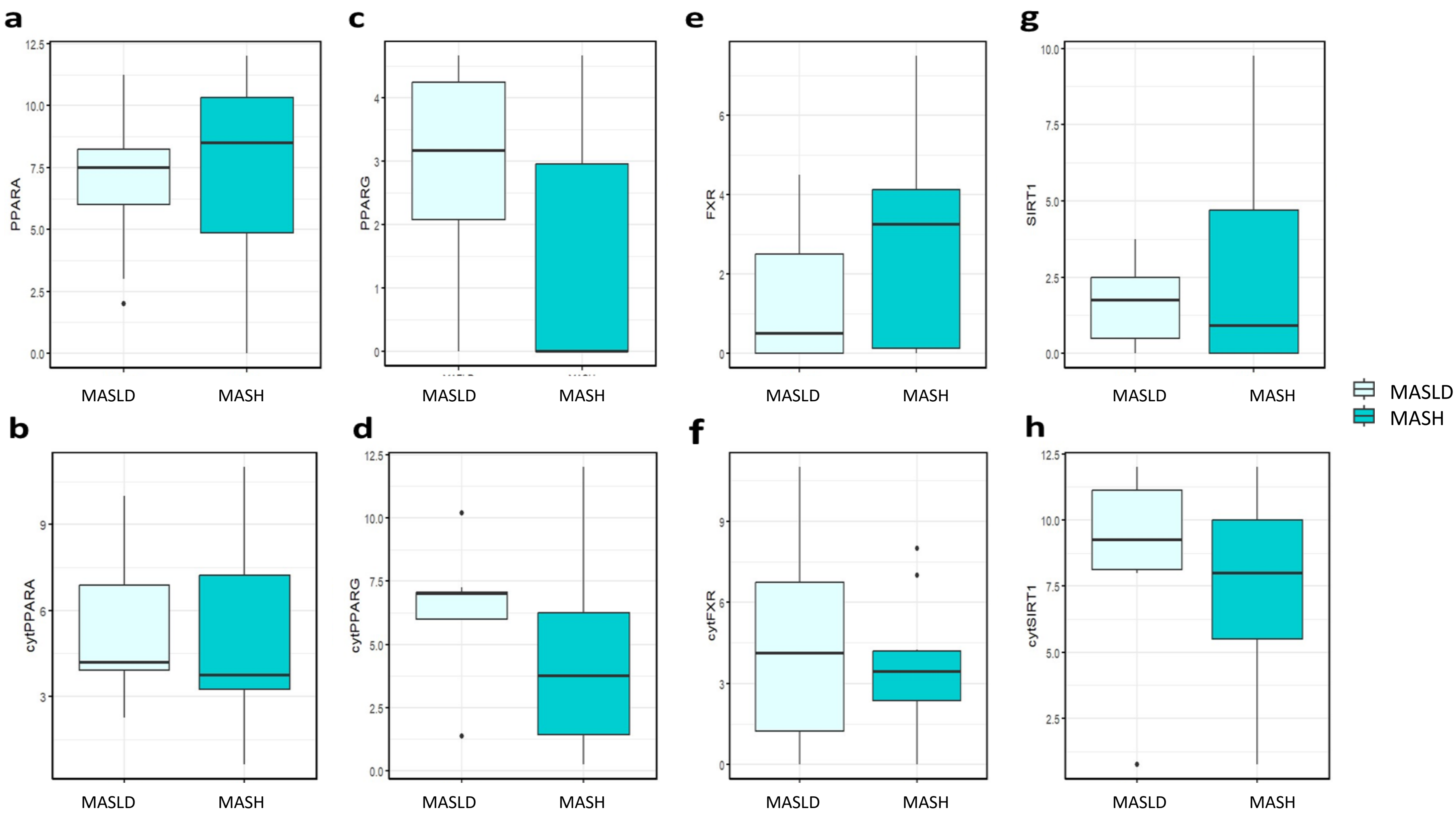

Supplementary Figure S2. Expression levels of PPARA, PPARG, FXR and SIRT1 between patients with MASLD and MASH using IHC scores. (a) Expression level of PPARA in patients with MASLD compared to patients with MASH (p = 0.5826). (b) Expression level of cytPPARA in patients with MASLD compared to patients with MASH (p = 0.5629). (c) Expression level of PPARG in patients with MASLD compared to patients with MASH (p = 0.0793). (d) Expression level of cytPPARG in patients with MASLD compared to patients with MASH (p = 0.1719). (e) Expression level of FXR in patients with MASLD compared to patients with MASH (p = 0.3514). (f) Expression level of cytFXR in patients with MASLD compared to patients with MASH (p = 0.7311). (g) Expression level of SIRT1 in patients with MASLD compared to patients with MASH (p = 0.9659). (h) Expression level of cytSIRT1 in patients with MASLD compared to patients with MASH (p = 0.6352). According to Normality test, Mann-Whitney test was employed.

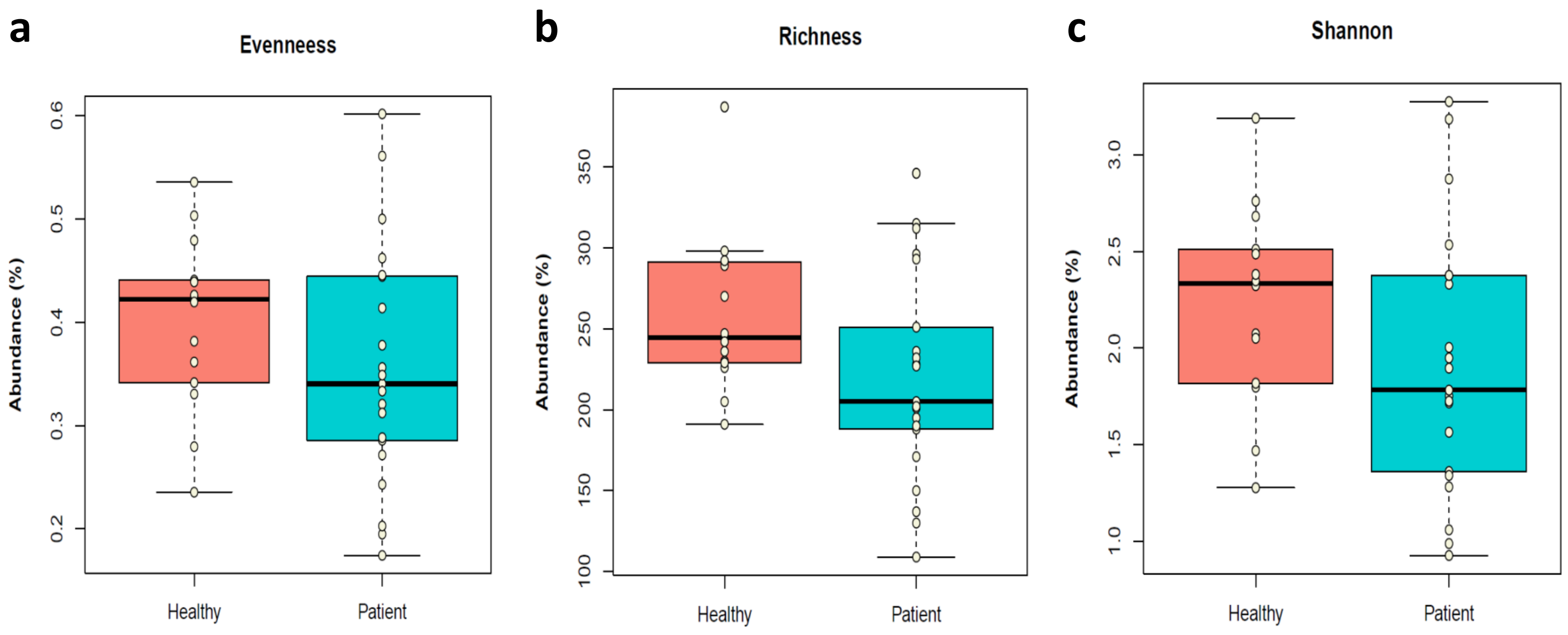

Supplementary Figure S3. Analysis of microbial evenness, richness and diversity between patients (Turquoise) and healthy controls (Salmon). a) Microbial evenness: no significant difference observed between patients and controls (Wilcoxon test *p*-value =0.1891). b) Microbial richness: no significant difference observed between patients and controls (Wilcoxon test *p*-value =0.064). c) Microbial diversity as measured by Shannon index: no significant difference observed between patients and controls (Wilcoxon test *p*-value =0.106).

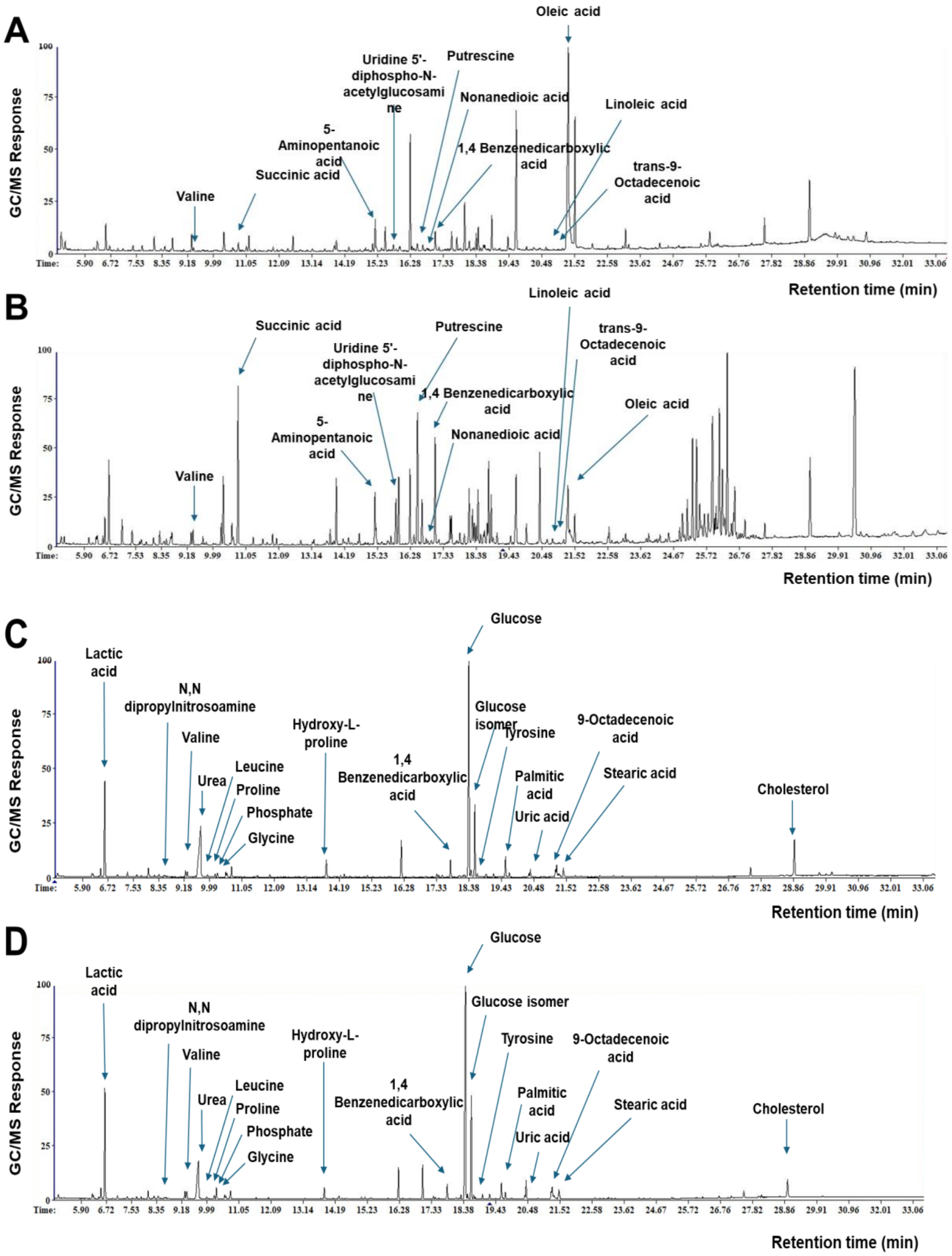

Supplementary Figure S4. Representative GC/MS chromatogram for compounds detected in MASLD patients and healthy control. (A) Representative chromatogram for compounds detected in fecal samples of healthy controls (B) Representative chromatogram for compounds detected in fecal samples of patients with MASLD. (C) Representative chromatogram for compounds detected in serum samples of healthy controls. (D) Representative chromatogram for compounds detected in serum samples of patients with MASLD. Metabolites altered among patients and controls are identified in representative chromatogram.

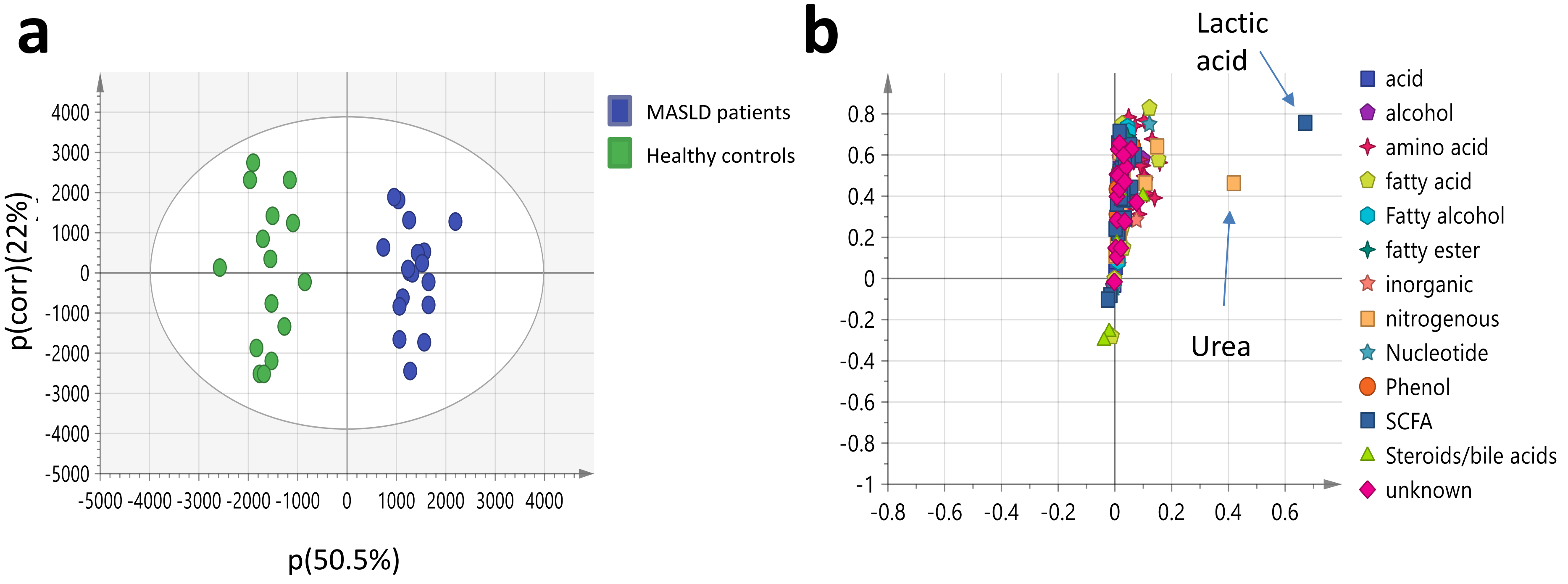

Supplementary Figure S5. GC/MS based serum metabolites OPLS of patients with MASLD (blue) versus healthy controls (green) after removal of sugars. (a) Score plot of serum metabolites OPLS (after removal of sugars) showing segregation between patients with MASLD (blue) and healthy controls (green) (R2 cum= 0.73, Q2 cum= 0.56 and *p*-value=0.003). (b) S-plot derived from serum metabolites OPLS model (after removal of sugars). showing the covariance p[1] against the correlation p(cor)[1] of the variables depicting contributing peaks with their assignment

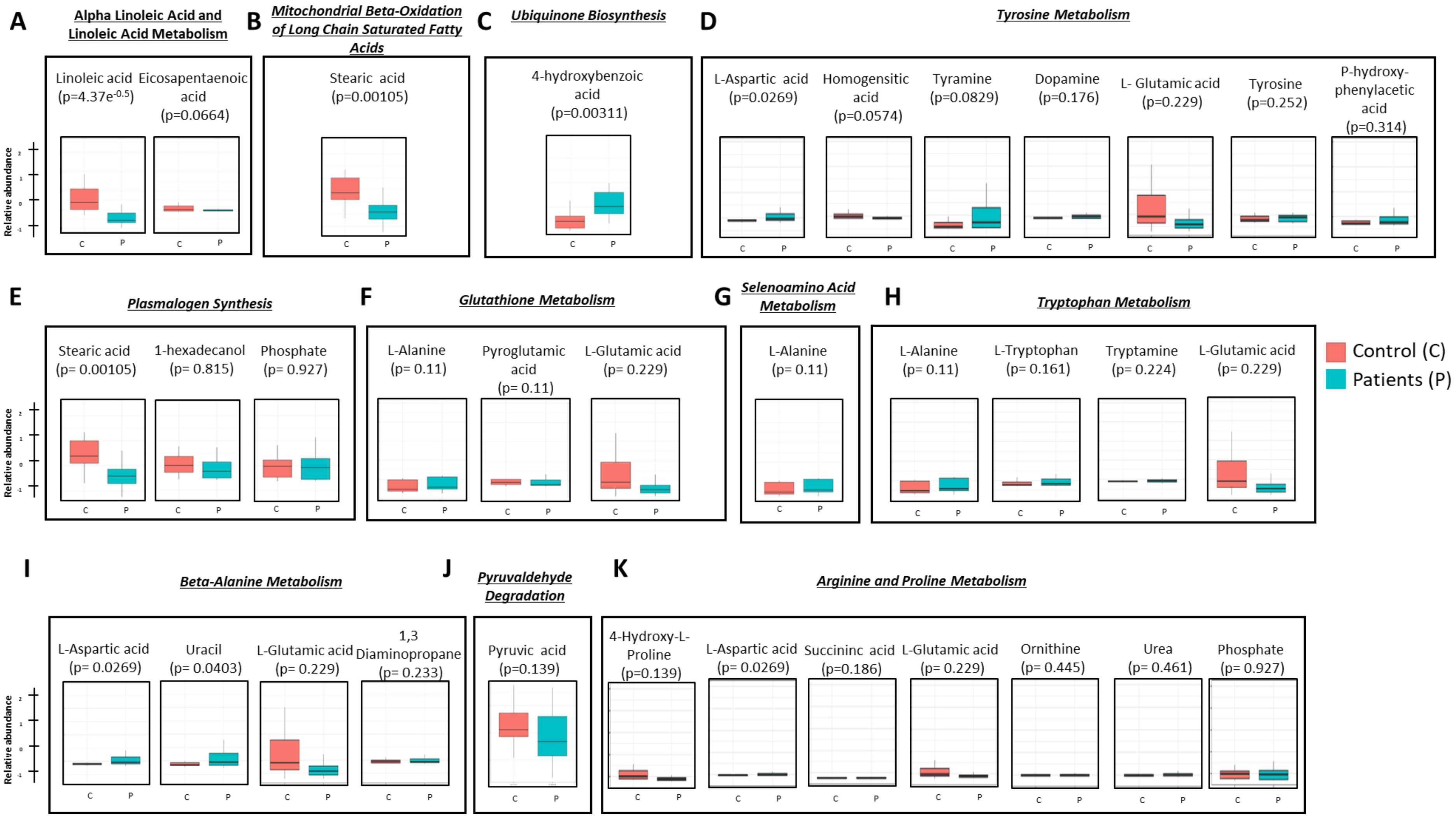

Supplementary Figure S6. Boxplots showing the top 11 enriched metabolic pathways with their most prominent fecal metabolites that are most significantly affected in patients with MASLD vs healthy controls. Turquoise bars represent patients, orange bars represent controls.

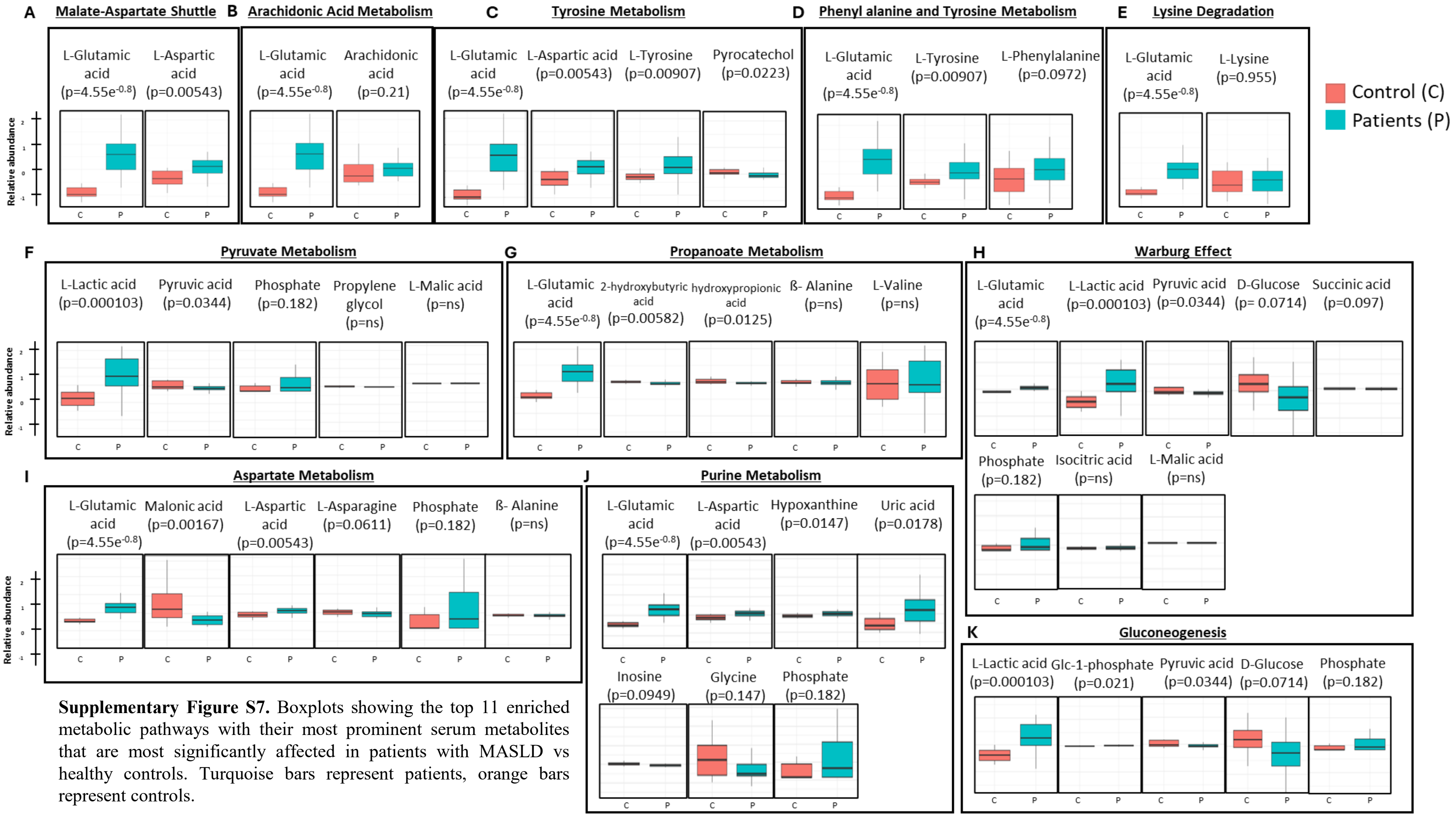

Supplementary Figure S7. Boxplots showing the top 11 enriched metabolic pathways with their most prominent serum metabolites that are most significantly affected in patients with MASLD vs healthy controls. Turquoise bars represent patients, orange bars represent controls.

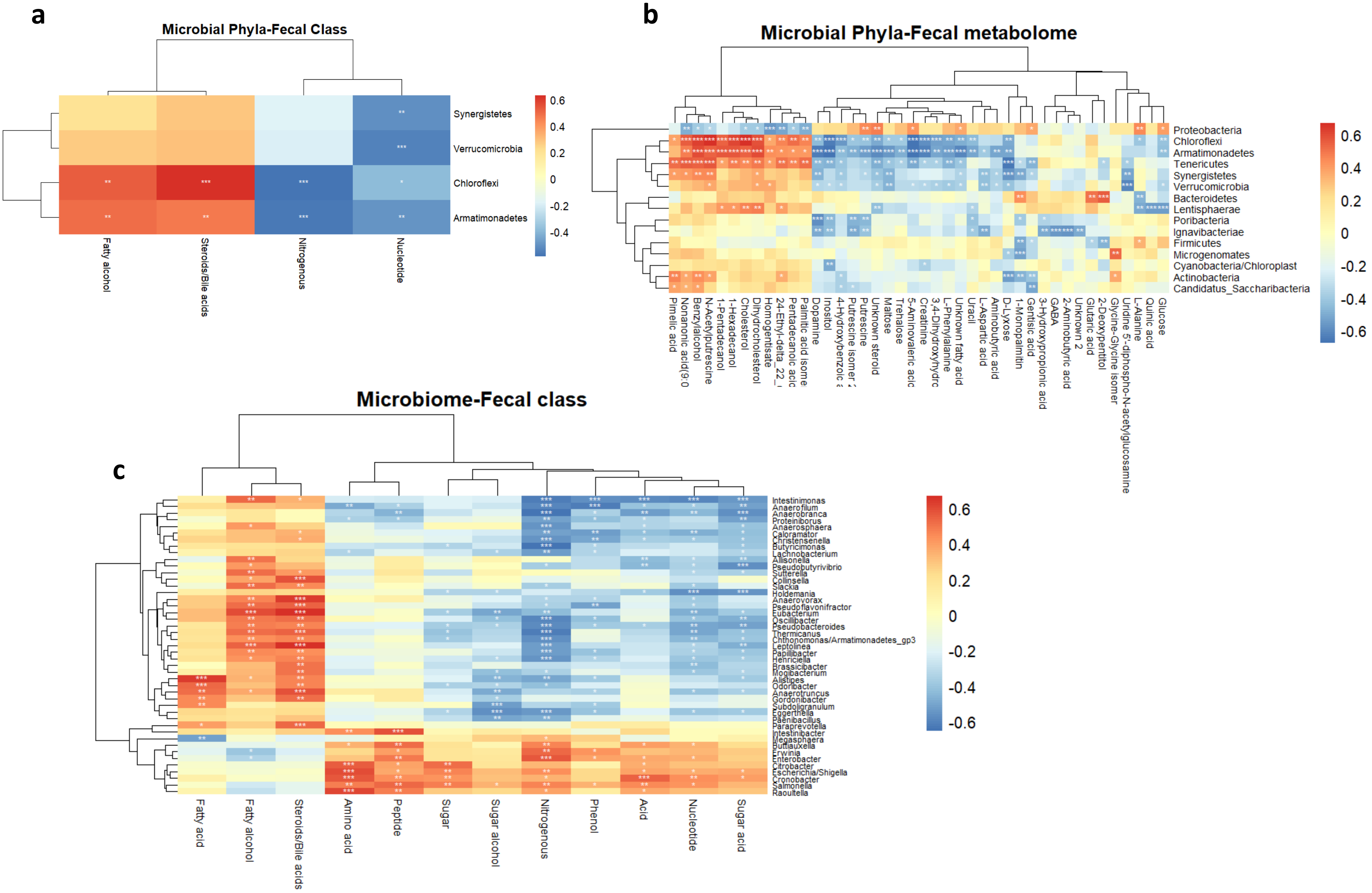

Supplementary Figure S8. Microbiome-fecal metabolome correlation coefficient analysis. a) Heatmap showing correlation between Microbial phyla and classes of fecal metabolites. b) Heatmap showing correlation between Microbial phyla and fecal metabolites. c) Heatmap showing correlation between Microbial genera and classes of fecal metabolites. Spearman correlation analysis was employed using pairwise method, heatmaps shows spearman correlation coefficient R cutoff of ±0.5 and corresponding statistically significant *p*-value. *p*-value less than 0.05 was considered statistically significant. *** = p <0.001, ** = p <0.01, * = p <0.05. Holm adjustment for multiple comparisons was applied.

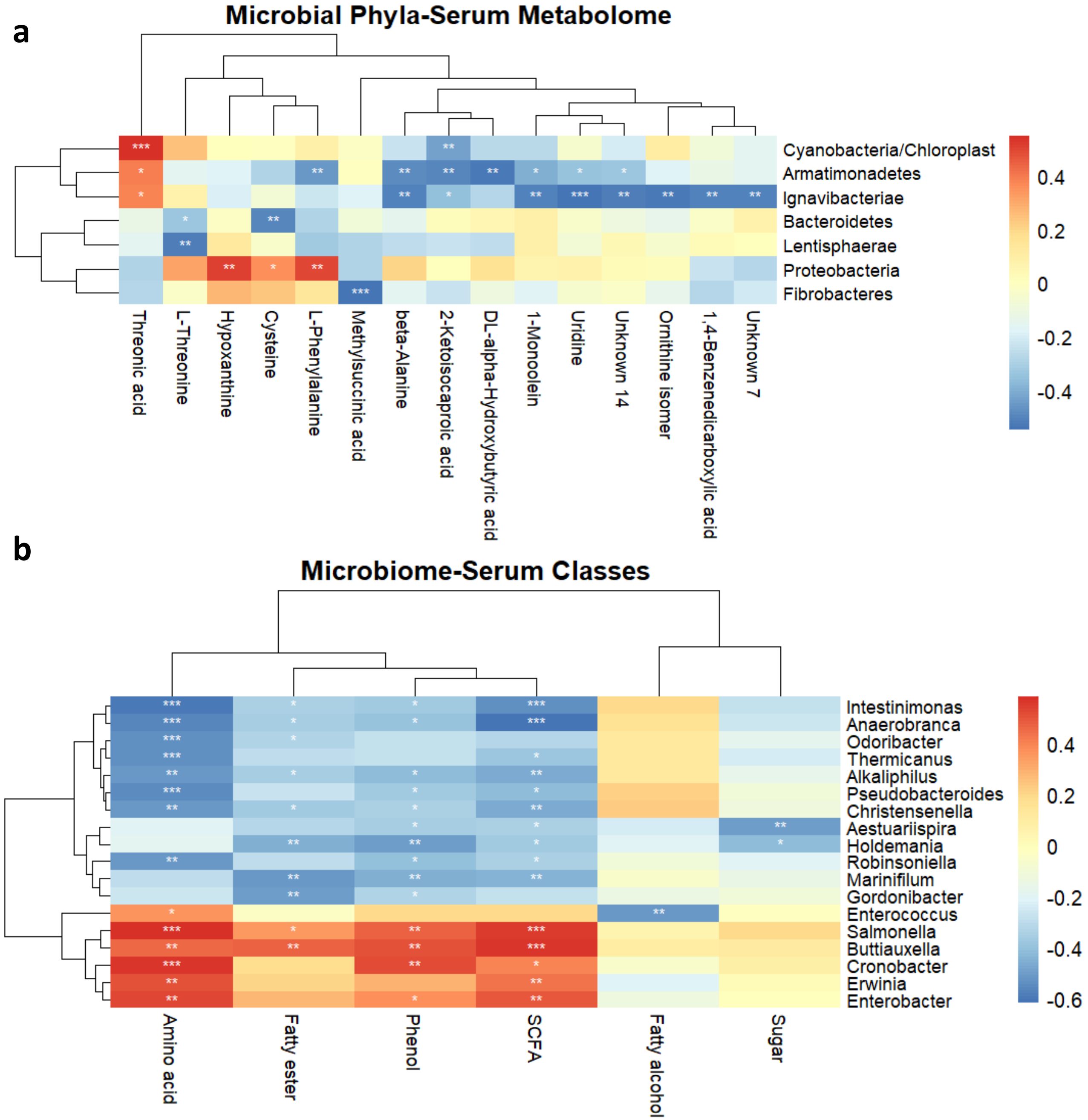

Supplementary Figure S9. Microbiome-serum metabolome correlation coefficient analysis. a) Heatmap showing correlation between Microbial Phyla and serum metabolites. b) Heatmap showing correlation between Microbial Genera and classes of serum metabolites. Spearman correlation analysis was employed using pairwise method, heatmaps shows spearman correlation coefficient R cutoff of ±0.5 and corresponding statistically significant *p*-value. *p*-value less than 0.05 was considered statistically significant. *** = p <0.001, ** = p <0.01, * = p <0.05. Holm adjustment for multiple comparisons was employed .

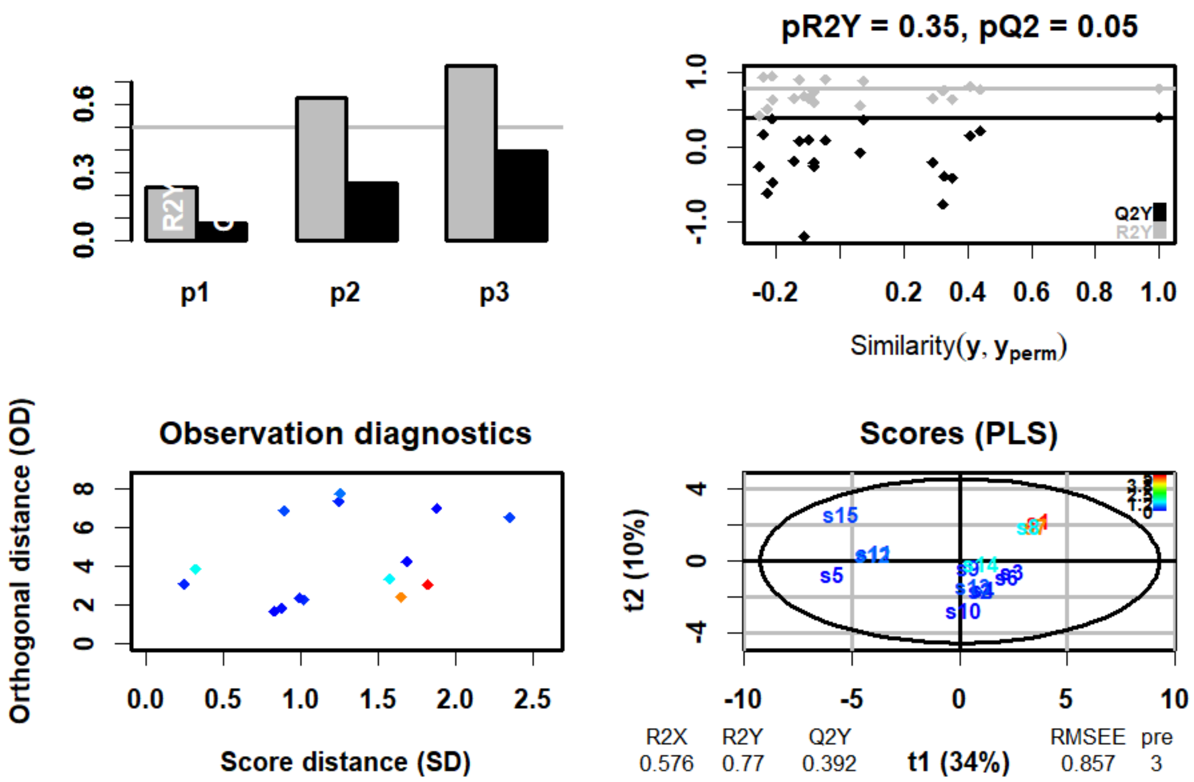

Supplementary Figure S10. Diagnostic analysis of SIRT1 PLS model. Top left: inertia barplot suggesting 3 orthogonal components that may be sufficient to capture most of the variation; Top right: significance diagnostic plot showing R^2Y and Q^2Y of the model when compared to the corresponding values obtained after y response (SIRT1 localization score) random permutation; Bottom left: observation diagnostics; Bottom right: score plot showing the number of components and the cumulative R^2X, R^2Y and Q^2Y below the plot.

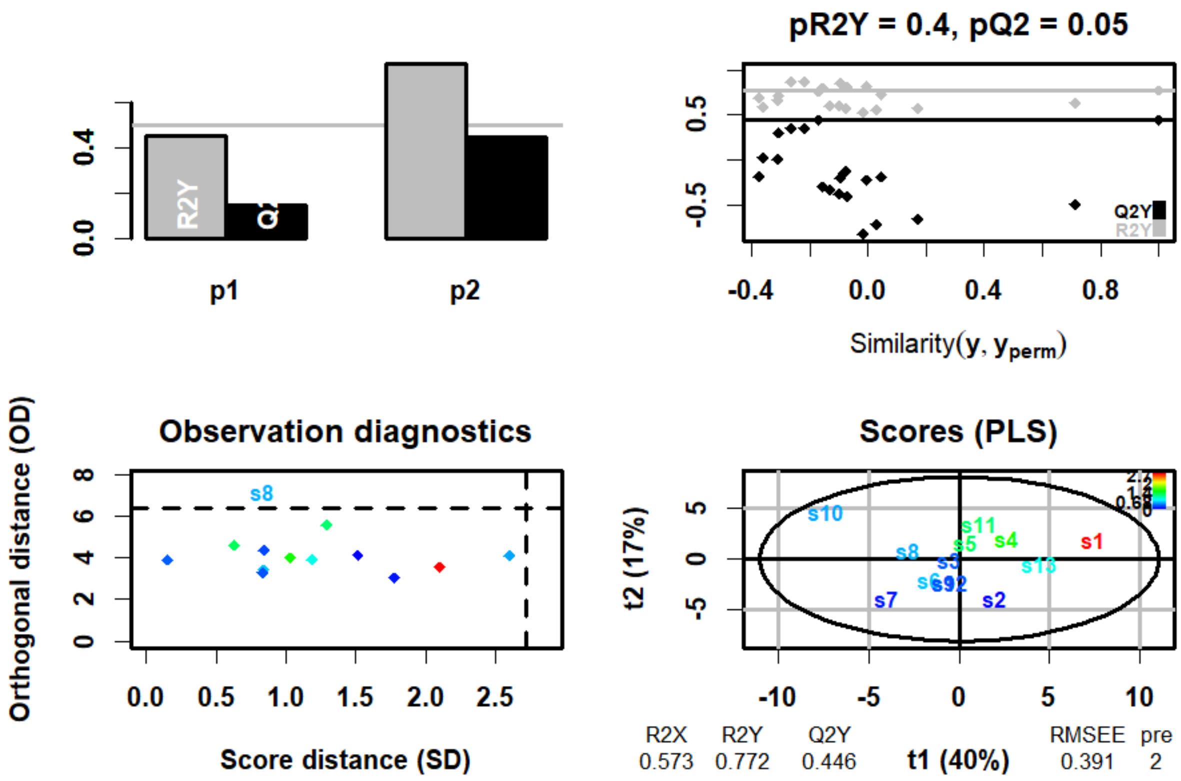

Supplementary Figure S11. Diagnostic analysis of FXR PLS model. Top left: inertia barplot suggesting 2 orthogonal components that may be sufficient to capture most of the variation; Top right: significance diagnostic plot showing R^2Y and Q^2Y of the model when compared to the corresponding values obtained after y response (FXR localization score) random permutation; Bottom left: observation diagnostics; Bottom right: score plot showing the number of components and the cumulative R^2X, R^2Y and Q^2Y below the plot.

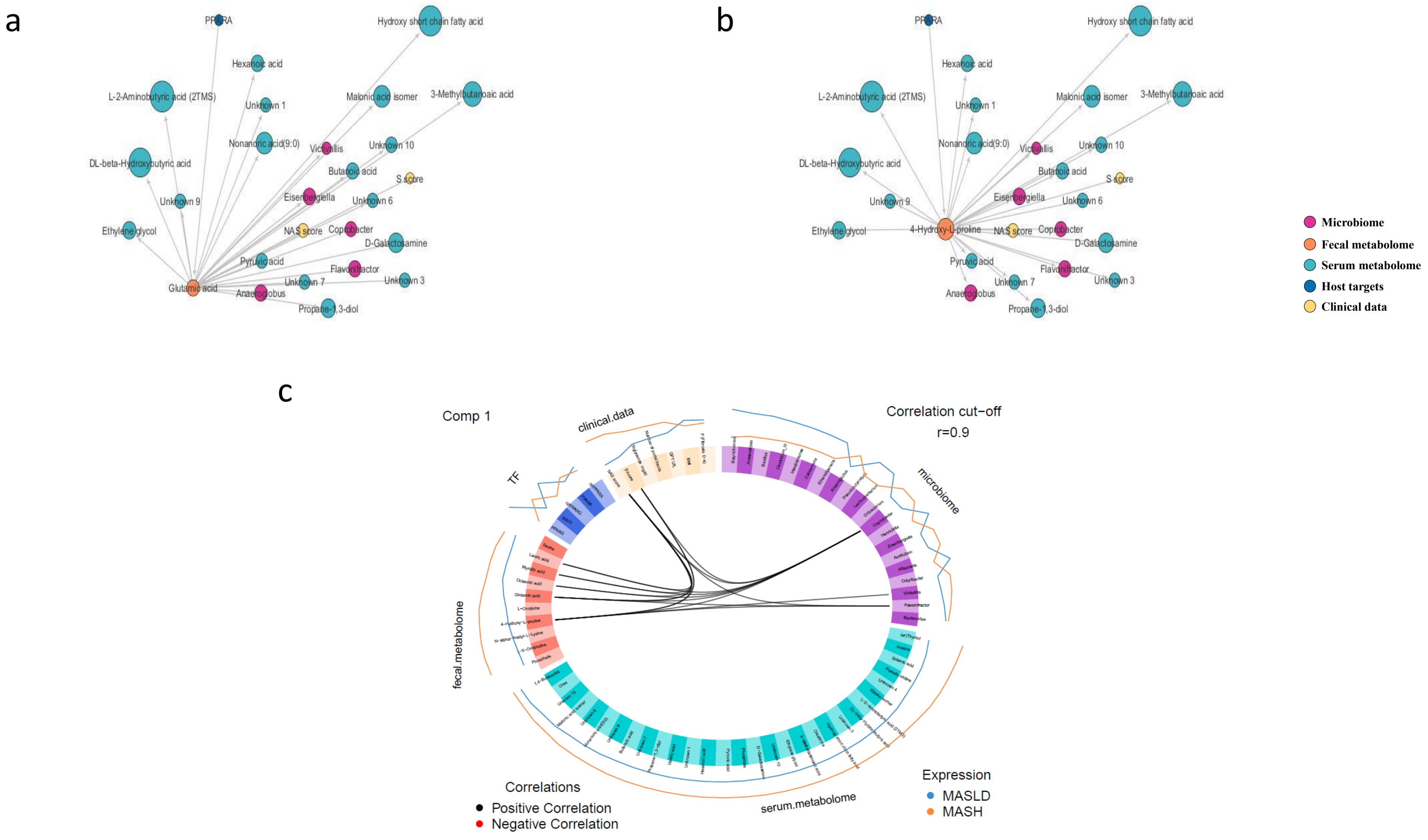

Supplementary Figure S12. DIABLO generated networks. a) Association subnetwork for glutamic acid as depicted by DIABLO model(r > 0.75). b) Association subnetwork for 4-hydroxy-L-proline as depicted by DIABLO model (r>0.75). Microbiome nodes colored in magenta, fecal metabolome nodes colored in orange, serum metabolome nodes colored in dark turquoise, host targets nodes colored in dark blue and clinical data nodes colored in yellow. Arrow head points towards target. c) Circos plot showing selected variables from DIABLO analysis and their correlations. The plot shows correlations more than 0.9 between the selected variables. Outer lines demonstrate expression levels of selected variables in each group (MASLD “blue” or MASH “orange”). Inner lines show correlations between variables (positive correlations “grey”, negative correlations “red”). Lines showing average expression levels of selected variables, illustrates that these variables can discriminate MASLD and MASH patients.
